# Supplementary material for: Pregnancy decisions after fetal or perinatal death: systematic review of qualitative research
Source: BMJ Open. 2019 Dec 23;9(12):e029930. doi: 10.1136/bmjopen-2019-029930 (PMC7008435; doi:10.1136/bmjopen-2019-029930)
Supplement: Supplementary data [file bmjopen-2019-029930supp001.pdf]

Appendix S1

	Search Terms
1.	(Stillbirth or still-birth or "perinatal loss" or "perinatal death" or "perinatal mortality" or "neonatal death" or miscarriage or "reproductive loss" or "pregnancy complications" or "fetal death" or "fetal resorption" or "foetal death" or "foetal resorption").mp.
2.	(Infant Mortality or Pregnancy outcome or Fetal Diseases or Foetal Diseases or Pregnancy Complications).mp.
3.	(baby death or newborn death or new-born death).mp.
4.	(abortion, spontaneous or abortion or “termination of pregnancy”).mp. [mp=title, abstract, original title, name of substance word, subject heading word, keyword heading word, protocol supplementary concept word, rare disease supplementary concept word, unique identifier, synonyms]
5.	(behavior or behaviour or emotion* or psychosocial or "emotional state" or "psychological state").mp. [mp=title, abstract, original title, name of substance word, subject heading word, keyword heading word, protocol supplementary concept word, rare disease supplementary concept word, unique identifier, synonyms]
6.	(bereavement or grief or expressed emotion).mp.
7.	(experience* or perception* or feeling* or attitude* or voice*).mp.
8.	(pregnancy or pre-pregnancy or "subsequent pregnancy" or "previous pregnancy" or "past pregnancy").mp.
9.	(pre-conception or preconception or "preconception care" or "prenatal care").mp.
10.	(peri-conception or periconception).mp.
11.	(interpregnancy or inter-pregnancy or "interpregnancy interval" or "inter-pregnancy interval").mp.
12.	("Birth Interval*" or "birth spacing").mp. [mp=title, abstract, original title, name of substance word, subject heading word, keyword heading word, protocol supplementary concept word, rare disease supplementary concept word, unique identifier, synonyms]
13.	(contraception or "contraception behavio*" or interconception or inter-conception or postpartum or post-partum).mp.
14.	(((Decision/ adj4 pregnanc*.mp.) or plan*.mp.) adj3 pregnancy.mp.) or "family planning".mp. [mp=title, abstract, original title, name of substance word, subject heading word, keyword heading word, protocol supplementary concept word, rare disease supplementary concept word, unique identifier, synonyms]
15.	(parent* or father or mother* or wom*n or "single parent" or spouse* or relationship* or "partner relationship*").mp. [mp=title, abstract, original title, name of substance word, subject heading word, keyword heading word, protocol supplementary concept word, rare disease supplementary concept word, unique identifier, synonyms]
16.	(psychosocial or psychological state).mp.
17.	(mixed-method or multi-method).mp. or empirical research/ or grounded theory/ or qualitative research/ or hermeneutics/ or interdisciplinary research/ or phenomenological research.mp. or phenomenology.mp. [mp=title, abstract, original title, name of substance word, subject heading word, keyword heading word, protocol supplementary concept word, rare disease supplementary concept word, unique identifier, synonyms]
18.	(interview or focus group or survey or questionnaire or diar* or cohort stud* or case stud* or ethnograph* or retrospective or longitudinal or observation*).mp. [mp=title, abstract, original title, name of substance word, subject heading word, keyword heading word, protocol supplementary concept word, rare disease supplementary concept word, unique identifier, synonyms]
19.	17 or 18
20.	1 or 2 or 3 or 4
21.	5 or 6 or 7 or 14 or 16
22.	8 or 9 or 10 or 11 or 12 or 13
23.	15 and 19 and 20 and 21 and 22
24.	limit 23 to (abstracts and english language and humans and "qualitative (maximizes sensitivity)")
25.	20
26.	limit 20 to abstracts
27.	15 and 19 and 21 and 22 and 26
28.	limit 27 to (english language and humans and "qualitative (maximizes specificity)")
