# Supplementary figures and images for: Pregnancy decisions after fetal or perinatal death: systematic review of qualitative research
Source: BMJ Open. 2019 Dec 23;9(12):e029930. doi: 10.1136/bmjopen-2019-029930 (PMC7008435; doi:10.1136/bmjopen-2019-029930)

## Appendix S2

PRISMA flow chart of search strategy and outcomes.

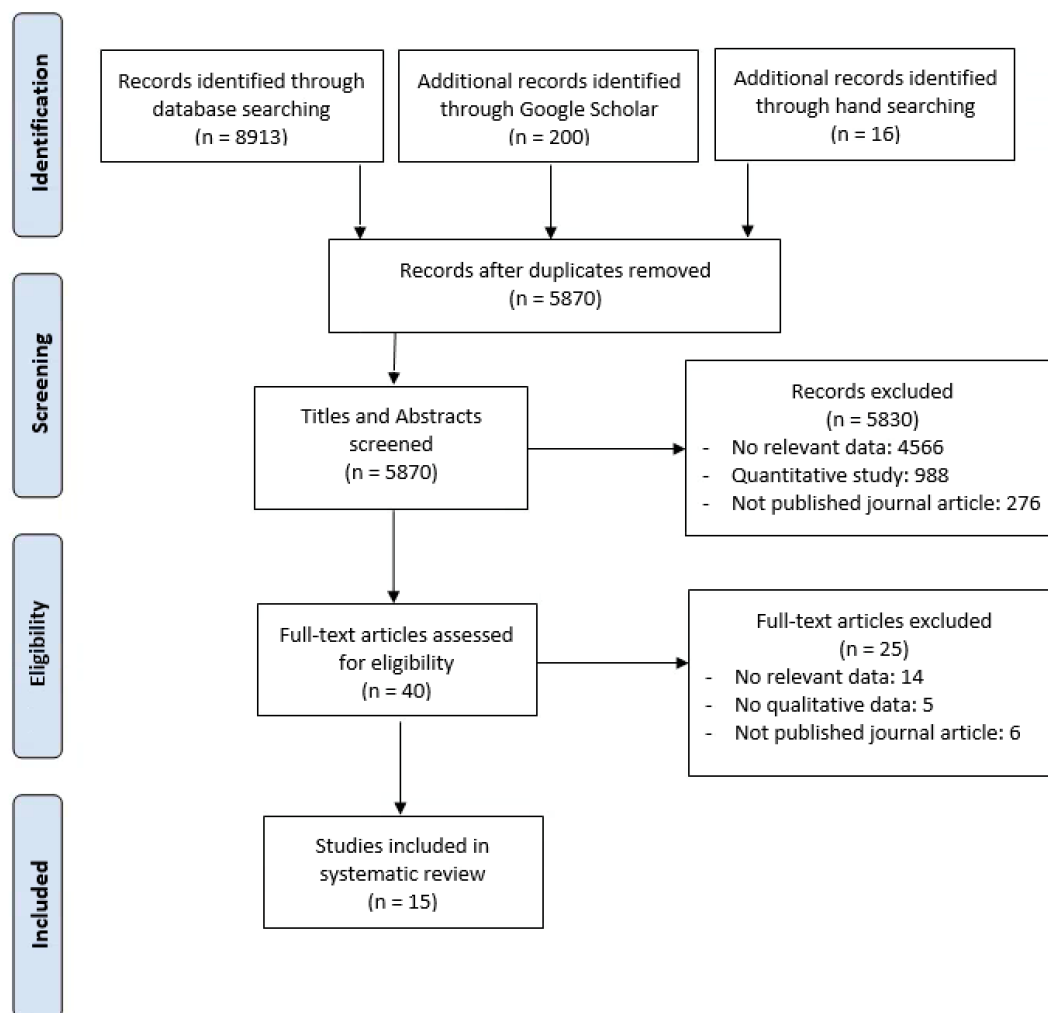

Supplement: Supplementary data [file bmjopen-2019-029930supp002.pdf]
