# Supplementary material for: Pregnancy decisions after fetal or perinatal death: systematic review of qualitative research
Source: BMJ Open. 2019 Dec 23;9(12):e029930. doi: 10.1136/bmjopen-2019-029930 (PMC7008435; doi:10.1136/bmjopen-2019-029930)
Supplement: Supplementary data [file bmjopen-2019-029930supp003.pdf]

Table S1 – Summary of Included Studies

Author (Year)	*Title*	*Sampling size and method*	*Data collection method*	*Data Analysis Method*	*Key characteristics of participants*	*Type of Loss*	*Retrospectivity of loss*	*Location*
Bansen and Stevens (1992)[52]	Women's experiences of miscarriage in early pregnancy	*n=10* Convenience sample	Open-ended interview	Content Analysis	- Mothers<br>- Aged: 23-33 (mean= 28.5 years)<br>- Ethnicity: white	Early miscarriage 6-15 weeks gestation (mean= 8.8 weeks)	2-5 months	USA
Cacciatore et al., (2008)[57]	When a baby dies: Ambiguity and stillbirth.	*n=74* Convenience sample	Focus group	Framework Analysis	- Grieving mothers, fathers, sisters, brothers, grandmothers, grandfathers and an aunt<br>- No demographic information	Stillbirth	Unknown	USA
Carlsson et al., (2016)[55]	Experiences of termination of pregnancy for fetal anomaly: A qualitative study of virtual community messages	*n= 122* Purposive sample	Cross-sectional study of messages in 2 virtual communities	Content Analysis	- Majority mothers (*n=112*)<br>- Experience of TOPFA in past year<br>- 106 (86.9%) had previous experience of TOPFA<br>- No demographic information	TOPFA after 15 weeks gestation	63% less than 1 year	Sweden
Cecil (1994)[53]	"I wouldn't have minded a wee one running about"	*n= 27* Convenience sample	Semi-structured interviews with follow up interviews	Content Analysis	- Mothers<br>- Aged 19-43<br>- Religion: Protestant (*n=13*), Catholic (*n=13*), No Religion (*n=1*)<br>- Limited demographic information	First trimester miscarriage	6 months	*UK*
Conway and Russell (2000)[54]	Couples' grief and experience of support in the aftermath of miscarriage	*n= 71* Purposive sample	Questionnaire	Content Analysis using coding frame	- Mothers (*n=39*) accompanied by partners (*n=32*)<br>-<br>- Previous children (mothers *n=22;* partners *n*=12)<br>- Socio-economic status tended towards average<br>- Limited demographic information	Miscarriage 5-16 weeks gestation (mean = 9.5 weeks)	2-4 months	*Australia*
Davis et al., (1989)[47]	Postponing Pregnancy after Perinatal Death: Perspectives on Doctor Advice	*n=24* Convenience sample	Open-ended interview	Frequency analysis	- Mothers<br>- Previous children (*n=24*)<br>- No demographic information	Stillbirth	1-10 years	*USA*
de Montigny et al., (1999)[56]	A baby has died: the impact of perinatal loss on family social networks	*n=20* Convenience sample	Open-ended questionnaire	Content analysis	- Mothers (*n=14)* and fathers (*n=6)*<br>-<br>- Experience of multiple losses: >1 perinatal death (*n=6);* 4 perinatal deaths (*n=2)*; 1 neonatal death (*n=1)*<br>- Ethnicity: white; French-speaking; middle class	Perinatal death	6 years	*Canada*

Author (Year)	*Title*	*Sampling size and method*	*Data collection method*	*Data Analysis Method*	*Key characteristics of participants*	*Type of Loss*	*Retrospectivity of loss*	*Location*
Grout and Romanoff (2000)[15]	The myth of the replacement child: parents' stories and practices after perinatal death	*n=10* Purposive sample	Open-ended interview	Content analysis	- Parents of 7 families (3 couples, 1 father, 3 mothers)<br>- Participants had a subsequent child between 3 and 5 years of age<br>- Experience of multiple losses (*n=3*)<br>- Previous children (*n=4*)<br>- Intact marriages<br>- Middle class; ethnicity: white	Stillbirth (including late-term miscarriage, or early infant death)	2-10 years	*USA*
Hsu et al., (2002)[50]	Transforming loss: Taiwanese women's adaption to stillbirth	n= 20 Purposive sample	Open-ended interview	Ethnographic Thematic Analysis	- Mothers<br>- Aged: 22-40 (mean = 30 years)<br>- First pregnancy (*n=6*)<br>- Previous children (*n=7*)<br>- Ethnicity: Taiwanese<br>- Limited demographic information	Stillbirth	0-2 years	*Taiwan*
Keim et al., (2017)[49]	Parent distress and the decision to have another child after an infant's death in the Neonatal Intensive Care Unit (NICU)	*n=69* Purposive sample	Semi-structured telephone interview	Content Analysis	- Mothers (*n=42*) and fathers (*n=27*) but technical difficulties meant interviews were analysed for mothers (*n=28*) and fathers (*n*=11)<br>- Aged: 18 years +<br>- Approximately 2/3 had subsequent child<br>- Majority married; majority completed secondary education<br>- Ethnicity: majority white; English speaking	Death of neonatal infant in NICU	3 months – 5 years	*USA*
Lee et al., (2013)[21]	Women's decision making and experience of subsequent pregnancy following stillbirth	*n=11* Convenience sample	Interview	Content Analysis	- Mothers<br>- No mothers had given birth to subsequent child but some were pregnant (*n=8*) (9-25 weeks (mean = 14 weeks)<br>- Aged: 26-43 (mean = 32 years)<br>- Majority first pregnancy (*n=8*)<br>- Married or cohabiting; majority in full-time work<br>- Ethnicity: white British (*n=8*), Slovakian (*n=1*), Italian (*n=1*), white Asian (*n=1*); English-speaking	Stillbirth: 24+ weeks' gestation (mean = 36 weeks)	6-12 months	*UK*

Author (Year)	*Title*	*Sampling size and method*	*Data collection method*	*Data Analysis Method*	*Key characteristics of participants*	*Type of Loss*	*Retrospectivity of loss*	*Location*
Meaney et al., (2017)[19]	Parents' concerns about future pregnancy after stillbirth: a qualitative study	*n=15*<br>Purposive sample	Semi-structured interview<br><br>Couples interviewed separately	Interpretative Phenomenological Analysis	- Mothers (*n=10*) and fathers (*n=5*)<br>- Irish<br>- No demographic information	Stillbirth	4-16 months	*Ireland*
Ockhuijsen et al., (2014)[48]	Pregnancy after miscarriage: balancing between loss of control and searching for control	*n=24*<br>Purposive sample	Semi-structured interview	Thematic Analysis	- Mothers<br>- Interviewed after 8 weeks of subsequent pregnancy. Those receiving fertility treatment were excluded<br>- Aged: 27-38 (mean=33 years)<br>- Prior experience of miscarriage 1-5 (median=2)<br>- Married or partnered; heterosexual<br>- Education ranged from secondary school to university<br>- Dutch speakers	Miscarriage	Unknown	*Netherlands*
Phipps (1986)[16]	The subsequent pregnancy after stillbirth: anticipatory parenthood in the face of uncertainty	*n=30*<br>Purposive sample	Open-ended interview	Content Analysis	- Couples interviewed together in all but one instance<br>- Experience of stillbirth (*n=8) or* neonatal death (*n=7*)<br>- Subsequent child aged 5months – 3 years (mean = 15 months)<br>- Mother mean age: 30.2 years; Father mean age: 33.7 years<br>- Previous children (*n*=6)<br>- Homogenous, middle class sample<br>- Intact, two-parent families<br>- Ethnicity: Black (*n=1*), Hispanic (*n*=1), Caucasian (*n=13*)	Neonatal death (including stillbirth due to prematurity or congenital anomalies)	Unknown	*USA*
Tseng et al., (2014)[51]	Taiwanese women's process of recovery from stillbirth: A qualitative descriptive study	*n=21*<br>Purposive sample	38 in-depth interview: face-to-face (*n=28*) and telephone (*n=4*)<br><br>11 participants took part in follow-up interviews	Thematic Analysis	- Mothers<br>- Aged: 23-43 (mean 31.9 years)<br>- Previous children (*n=9*)<br>- Married<br>- Employed (*n=14*)<br>- Ethnicity: Taiwanese<br>- Religion: Taiwanese folk beliefs (*n=8*), Buddhism (*n=4*), Taoism (*n=3*), Christianity (*n=2*), I-Kuan Tao (*n=2*)	Stillbirth (20-37 weeks gestation)	6 months – 6 years (mean = 2.3 years)	*Taiwan*

TOPFA – termination of pregnancy for fetal anomaly

Dyer E, *et al. BMJ Open* 2019; 9:e029930. doi: 10.1136/bmjopen-2019-029930
