# Supplementary material for: Pregnancy decisions after fetal or perinatal death: systematic review of qualitative research
Source: BMJ Open. 2019 Dec 23;9(12):e029930. doi: 10.1136/bmjopen-2019-029930 (PMC7008435; doi:10.1136/bmjopen-2019-029930)
Supplement: Supplementary data [file bmjopen-2019-029930supp004.pdf]

Table S2: CASP Quality Assessment Results

CASP Question	Bansen and Stevens (1992)[52]	Cacciatore *et al.,* (2008)[57]	Carlsson *et al.,* (2016)[55]	Cecil (1994)[53]	Conway and Russell (2000)[54]	Davis *et al.,* (1989)[47]	de Montigny *et al.,* (1999)[56]	Grout and Romanoff (2000)[15]	Hsu *et al.,* (2002)[50]	Keim *et al.,* (2017)[49]	Lee *et al.,* (2013)[21]	Meaney *et al.,* (2017)[19]	Ockhuijsen *et al.,* (2014)[48]	Phipps (1986)[16]	Tseng *et al.,* (2014)[51]
1	Y	Y	Y	Y	Y	Y	Y	Y	Y	Y	Y	Y	Y	Y	Y
2	Y	Y	Y	Y	Y	Y	Y	Y	Y	Y	Y	Y	Y	Y	Y
3	Y	Y	Y	Y	Y	C	Y	Y	C	Y	Y	Y	Y	Y	Y
4	Y	Y	Y	Y	Y	C	Y	Y	C	Y	Y	C	Y	Y	Y
5	Y	C	Y	C	Y	C	Y	Y	C	Y	Y	Y	Y	Y	Y
6	Y	N	Y	N	N	N	N	C	Y	Y	C	N	N	C	N
7	N	N	Y	C	C	N	C	C	Y	Y	Y	Y	Y	N	Y
8	Y	N	Y	C	C	C	Y	C	C	Y	Y	Y	Y	C	Y
9	Y	N	Y	Y	Y	Y	Y	Y	Y	Y	Y	Y	Y	Y	Y
10	Y	Y	Y	N	Y	Y	Y	Y	Y	Y	Y	Y	Y	Y	Y
**CASP Score**	18	11	20	13	16	12	17	17	16	20	19	17	18	16	18
**Quality**	Good	Average	Good	Average	Good	Average	Good	Good	Good	Good	Good	Good	Good	Good	Good

Key:
Y – Yes = 2
C - Can't Tell = 1
N- No = 0
