# Supplementary material for: Pregnancy decisions after fetal or perinatal death: systematic review of qualitative research
Source: BMJ Open. 2019 Dec 23;9(12):e029930. doi: 10.1136/bmjopen-2019-029930 (PMC7008435; doi:10.1136/bmjopen-2019-029930)
Supplement: Supplementary data [file bmjopen-2019-029930supp005.pdf]

Table S3 – Themes and subthemes emerging from the included studies

Author (year)	Deciding about becoming pregnant		Diversity of personal reactions			Social network influences		Planning or timing subsequent pregnancy		
	Immediacy of thoughts	*Differences in reactions between parents*	*Emotional response to loss*	*Imagined family*	*Ease perinatal grief*	*Altered relationships and social networks*	*Cultural norms and expectations*	*Barriers*	*Health professional advice*	*Health professional information and reassurance*
Bansen and Stevens (1992)[52]			✓			✓	✓			✓
Cacciatore et al., (2008)[57]		✓	✓	✓		✓		✓		
Carlsson et al., (2016)[55]	✓		✓	✓	✓	✓	✓	✓		✓
Cecil (1994)[53]	✓	✓	✓				✓			
Conway and Russell (2000)[54]		✓	✓						✓	
Davis et al., (1989)[47]	✓		✓	✓	✓			✓	✓	✓
de Montigny et al., (1999)[56]			✓			✓	✓		✓	✓
Grout and Romanoff (2000)[15]			✓	✓						
Hsu et al., (2002)[50]	✓		✓	✓		✓	✓			
Keim et al., (2017)[49]	✓	✓	✓	✓	✓			✓	✓	✓
Lee et al., (2013)[21]	✓		✓	✓	✓					✓
Meaney et al., (2017)[19]	✓	✓	✓	✓		✓	✓		✓	✓
Ockhuijsen et al., (2014)[48]			✓	✓		✓	✓		✓	
Phipps (1986)[16]		✓	✓	✓		✓	✓	✓	✓	✓
Tseng et al., (2014)[51]	✓		✓	✓	✓		✓			
