# Supplementary material for: Pregnancy decisions after fetal or perinatal death: systematic review of qualitative research
Source: BMJ Open. 2019 Dec 23;9(12):e029930. doi: 10.1136/bmjopen-2019-029930 (PMC7008435; doi:10.1136/bmjopen-2019-029930)
Supplement: Supplementary data [file bmjopen-2019-029930supp006.pdf]

ENTREQ Reporting Template

Number	Item	Description	Page/Line Number
1	**Aim**	State the research question the synthesis addresses.	Page 3, line 87-89
2	**Synthesis methodology**	Identify the synthesis methodology or theoretical framework which underpins the synthesis, and describe the rationale for choice of methodology *(e.g. meta-ethnography, thematic synthesis, critical interpretive synthesis, grounded theory synthesis, realist synthesis, meta-aggregation, meta-study, framework synthesis).*	Page 5, lines 131-147
3	**Approach to searching**	Indicate whether the search was pre-planned (comprehensive search strategies to seek all available studies) or iterative (to seek all available concepts until they theoretical saturation is achieved).	Page 3, lines 93-94<br>Page 4, lines 95-108
4	**Inclusion criteria**	Specify the inclusion/exclusion criteria *(e.g. in terms of population, language, year limits, type of publication, study type).*	Page 3, lines 93-94<br>Appendix S1
5	**Data sources**	Describe the information sources used (e.g. *electronic databases (MEDLINE, EMBASE, CINAHL, psycINFO, Econlit), grey literature databases (digital thesis, policy reports), relevant organisational websites, experts, information specialists, generic web searches (Google Scholar) hand searching, reference lists)* and when the searches conducted; provide the rationale for using the data sources.	Page 4, lines 100-102
6	**Electronic search strategy**	Describe the literature search *(e.g. provide electronic search strategies with population terms, clinical or health topic terms, experiential or social phenomena related terms, filters for qualitative research, and search limits).*	Page 3, lines 93-94<br>Page 4 95-98
7	**Study screening methods**	Describe the process of study screening and sifting *(e.g. title, abstract and full text review, number of independent reviewers who screened studies).*	Page 4, lines 102-108
8	**Study characteristics**	Present the characteristics of the included studies *(e.g. year of publication, country, population, number of participants, data collection, methodology, analysis, research questions).*	Page 5, lines 155-161<br>Page 6, lines 162-163
9	**Study selection results**	Identify the number of studies screened and provide reasons for study exclusion *(e,g, for comprehensive searching, provide numbers of studies screened and reasons for exclusion indicated in a figure/flowchart; for iterative searching describe reasons for study exclusion and inclusion based on modifications t the research question and/or contribution to theory development).*	Appendix S2<br><br>Page 13, lines 418-419
10	**Rationale for appraisal**	Describe the rationale and approach used to appraise the included studies or selected findings *(e.g. assessment of conduct (validity and robustness), assessment of reporting (transparency), assessment of content and utility of the findings).*	Page 4, lines 111-122
11	**Appraisal terms**	State the tools, frameworks and criteria used to appraise the studies or selected findings *(e.g. Existing tools: CASP, QARI, COREQ, Mays and Pope; reviewer developed tools; describe the domains assessed: research team, study design, data analysis and interpretations, reporting).*	Page 4, lines 111-122
12	**Appraisal process**	Indicate whether the appraisal was conducted independently by more than one reviewer and if consensus was required.	Page 4, lines 115-116
13	**Appraisal results**	Present results of the quality assessment and indicate which articles, if any, were weighted/excluded based on the assessment and give the rationale.	Table S2
14	**Data Extraction**	Indicate which sections of the primary studies were analysed and how were the data extracted from the primary studies? *(e.g. all text under the headings*	Page 4, lines 124-127

		"results /conclusions" were extracted electronically and entered into a computer software).	Page 5, lines 128-129
**15**	**Software**	State the computer software used, if any.	Page 4, line 102<br>Page 5, line 129
**16**	**Number of reviewers**	Identify who was involved in coding and analysis.	Page 5, line 132
**17**	**Coding**	Describe the process for coding of data *(e.g. line by line coding to search for concepts).*	Page 4, lines 134-147
**18**	**Study comparison**	Describe how were comparisons made within and across studies *(e.g. subsequent studies were coded into pre-existing concepts, and new concepts were created when deemed necessary).*	Page 5, lines 136-137
**19**	**Derivation of themes**	Explain whether the process of deriving the themes or constructs was inductive or deductive.	Page 5, lines 138-147
**20**	**Quotations**	Provide quotations from the primary studies to illustrate themes/constructs, and identify whether the quotations were participant quotations of the author's interpretation.	Page 6, lines 184-185<br>Page 7, lines 197-201<br>Page 8, lines 227-228 / 236-237 / 244-245<br>Page 9, lines 274-290<br>Page 10, lines 299-302<br>Page 11, line 331-333 / 346-351<br>Page 12, line 365 / 378-380
**21**	**Synthesis Output**	Present rich, compelling and useful results that go beyond a summary of the primary studies (e.g. *new interpretation, models of evidence, conceptual models, analytical framework, development of a new theory or construct).*	Figure 1<br>Discussion pages 13-16
