# Supplementary material for: Pregnancy decisions after fetal or perinatal death: systematic review of qualitative research
Source: BMJ Open. 2019 Dec 23;9(12):e029930. doi: 10.1136/bmjopen-2019-029930 (PMC7008435; doi:10.1136/bmjopen-2019-029930)
Supplement: Author's manuscript [file bmjopen-2019-029930.draft_revisions.pdf]

BMJ Open is committed to open peer review. As part of this commitment we make the peer review history of every article we publish publicly available.

When an article is published we post the peer reviewers' comments and the authors' responses online. We also post the versions of the paper that were used during peer review. These are the versions that the peer review comments apply to.

The versions of the paper that follow are the versions that were submitted during the peer review process. They are not the versions of record or the final published versions. They should not be cited or distributed as the published version of this manuscript.

BMJ Open is an open access journal and the full, final, typeset and author-corrected version of record of the manuscript is available on our site with no access controls, subscription charges or pay-per-view fees (http://bmjopen.bmj.com).

If you have any questions on BMJ Open's open peer review process please email info.bmjopen@bmj.com

BMJ Open

Pregnancy decisions after fetal or perinatal death: systematic review of qualitative research

Journal:	*BMJ Open*
Manuscript ID	bmjopen-2019-029930
Article Type:	Research
Date Submitted by the Author:	20-Feb-2019
Complete List of Authors:	Dyer, Ella; University of Newcastle, Institute of Health & Society<br>Bell, Ruth; University of Newcastle, Institute of Health & Society<br>Graham, Ruth; Newcastle University, School of Geography, Sociology and Politics<br>Rankin, Judith; Newcastle University, Institute of Health & Society
Keywords:	MENTAL HEALTH, OBSTETRICS, PERINATOLOGY, QUALITATIVE RESEARCH

SCHOLARONE™
Manuscripts

Title: Pregnancy decisions after fetal or perinatal death: systematic review of qualitative research

Authors: Ella Dyer[1], Ruth Bell[1], Ruth Graham[2], Judith Rankin[1]

1. Institute of Health & Society
   Newcastle University
   Baddiley-Clark Building
   Richardson Road
   Newcastle upon Tyne

2. School of Geography, Politics and Sociology
   Newcastle University
   19 Windsor Terrace
   Newcastle upon Tyne
   NE1 7RU

Corresponding author: Professor Judith Rankin

Contact details:

Address:

Institute of Health & Society

Newcastle University

Baddiley-Clark Building

Richardson Road

Newcastle upon Tyne

NE2 4AX

Telephone number: +44 191 208 5267

Email: Judith.rankin@ncl.ac.uk

Running title: Pregnancy decisions after loss: qualitative systematic review

For peer review only - http://bmjopen.bmj.com/site/about/guidelines.xhtml

Abstract

Objectives To synthesise the findings of qualitative research exploring parents' experiences, views and decisions about becoming pregnant following a perinatal death or fetal loss.

Design: Systematic review and meta-synthesis of qualitative research

Inclusion criteria: Nine electronic databases were searched using predefined search terms. Articles published in English, in peer reviewed journals, using qualitative methods to explore the experiences and attitudes of bereaved parents following perinatal or fetal loss, were included.

Data collection and analysis Qualitative data relating to first order and second order constructs were extracted and synthesised across studies using a thematic analysis.

Main results 15 studies were included. Four descriptive themes and 10 subthemes were identified. The descriptive themes were: deciding about subsequent pregnancy; individual reaction to the event; social network responses, and planning or timing of subsequent pregnancy. The decision to become pregnant after death is complex and varies between individuals and sometimes within couples. Decisions are often made quickly, in the immediate aftermath of a pregnancy loss, but may evolve over time. Bereaved parents may feel isolated from social networks.

Conclusion There is an opportunity to support parents to prepare for a pregnancy after a fetal or perinatal loss, and conversations may be welcomed at an early stage. Health professionals may play an important role providing support lacking from usual social networks.

PROSPERO registration: CRD42018112839

Strengths and limitations of this study:

- This review synthesised qualitative data from primary studies describing the experiences of bereaved parents in their transition from perinatal death to pregnancy
- We used standardised methods, including double blind screening, quality rating and data extraction
- The themes identified were supported by evidence across all included studies
- Study participants were mainly mothers from high income countries, with high levels of education and living with their partners, limiting the wider applicability of the findings
- The review was limited to peer-reviewed journal articles published in English

For peer review only - http://bmjopen.bmj.com/site/about/guidelines.xhtml

Introduction

Perinatal and fetal death remain common adverse pregnancy outcomes[1, 2], with a perinatal mortality rate of 6.6 per 1000 births in England and Wales[3], and estimated miscarriage rate of 25%-43%[4, 5]. Many parents who have experienced fetal or perinatal loss will have further pregnancies. Debate continues about recommendations concerning the optimum inter-pregnancy interval following such a death[6-9]. The WHO recommends couples wait at least six months before trying to conceive again[10], based on evidence that shorter inter-pregnancy intervals are associated with adverse pregnancy outcome [9, 11-13]. However, a recent meta-analysis found no clear evidence to support this recommendation[9], and UK guidance does not specify a waiting period. An additional concern is allowing sufficient time to grieve, and minimise the risk of trying to replace the deceased child, both of which have been associated with psychological and bonding issues [14,15, 16]. Hence, parents may receive contradictory advice from health professionals[17].

Many women experience an overwhelming urge to become pregnant as soon as possible after fetal or perinatal death [18-20]; 80% of women become pregnant within 18 months of the death[20-24]. The motives and processes involved in subsequent pregnancy decisions remain unclear. Health professional involvement in the decision to conceive is encouraged, to support women where modifiable risks can be reduced to try and avert subsequent perinatal death[25, 26]. However, many women only inform health professionals once they have become pregnant[27-29], suggesting that the consideration of conception is primarily a personal decision between partners[19].

This systematic review aimed to identify, appraise and summarise existing qualitative research reporting parents' experience of the decision-making process concerning becoming pregnant again after experiencing fetal or perinatal death.

Methods

Search strategy

Qualitative research reporting bereaved parents' inter-pregnancy experiences pertaining to thinking about, planning or preparing for subsequent pregnancy following a perinatal death

For peer review only - http://bmjopen.bmj.com/site/about/guidelines.xhtml

(miscarriage, stillbirth, termination of pregnancy for fetal anomaly (TOPFA) or neonatal death) was eligible for inclusion, if published in English in a peer-reviewed journal article. Search terms were identified using the SPIDER framework (Sample, Phenomenon of Interest, Design, Evaluation, Research Type)[30-32] (Appendix S1).

Electronic database searches (Medline, Web of Science, CINAHL, PsycINFO, ASSIA, Embase, PUBMED, Scopus and Google Scholar) were conducted between February and June 2018.

Titles and abstracts were screened by ED using the web-based tool 'Rayyan'[33]. A 10% sample was blindly double screened, and any title highlighted as potentially relevant by either reviewer included in the full-text review. Two reviewers independently read all full text articles. Uncertainties about inclusion were resolved through discussion with a third reviewer. All included studies were also manually citation searched.[34-39] [40] Fifteen studies were included in the final review (Appendix S2).

Quality appraisal

Study quality was assessed using the 2018 Critical Appraisal Skills Programme (CASP) checklist for qualitative research[41]. Numerical values were assigned to the three possible answers to the CASP questions (yes = 2, can't tell = 1, no = 0), with a maximum possible score of 20. Studies were considered 'good quality' if the overall score was 16 or more. Two reviewers independently rated each study, and differences were resolved by discussion. The quality score was considered during data analysis to ensure that all themes were present in better quality studies.

Data extraction and synthesis

Data including bibliographic information, aims, method, quality assessment and findings were extracted onto an Excel form. Qualitative data relating to first order (participants' quotes) and second order constructs (researcher interpretations, assumptions, statements and ideas) were recorded and imported into QSR NVIVO 12 for management and thematic analysis.

Thematic analysis techniques, adapted from Butler *et al.,*[38] and Harden and Thomas[42], were used to systematically analyse and synthesise qualitative data from the included studies in two iterative stages:

For peer review only - http://bmjopen.bmj.com/site/about/guidelines.xhtml

Stage 1: Coding text: data from the primary studies were examined for meaning and content, and within each study conceptual codes were systematically assigned allowing emerging concepts to be aggregated across the studies.
Stage 2: Development of descriptive themes: the list of codes was examined and re-analysed for meaning and organised into categories. Each category was examined and descriptive themes were organised to reveal relationships and link theoretically similar sub-themes together.

A priori themes of interest included: parents' decisions about pregnancy subsequent to loss, experiences surrounding preconception preparation and conception in the context of perinatal death, and the role of health professionals. All themes were reviewed by a second researcher.

Results

Included studies

Fifteen studies published between 1986 and 2017 and conducted in eight countries met the inclusion criteria (Appendix S2, Table S1). Sample sizes ranged from four to 122 participants, and fetal or perinatal deaths occurred across a range of gestations. Seven studies focused solely on women's experiences[21, 43-48,] seven included both parents[15, 16, 19, 49-52] and one included wider family members. Participants predominantly reported ethnicity as white[15, 16, 21, 46, 51, 52], relationship status as married or cohabiting[15, 16, 19, 21, 44, 45, 47-49, 51] and had completed some form of post-secondary education[16, 48, 49, 51]. Twelve studies were considered 'good quality', including details of ethical approval, participant recruitment, research design, and evidence of researcher reflexivity (Table S2).

Findings

There were four main descriptive themes: 1) Deciding about subsequent pregnancy; 2) Personal reaction to event; 3) Social network responses; 4) Planning or timing of subsequent pregnancy. Ten subthemes were identified which interacted with the four themes (figure 1). Themes and subthemes were tabulated according to the studies from which they arose (Table S3).

For peer review only - http://bmjopen.bmj.com/site/about/guidelines.xhtml

Theme one: deciding about becoming pregnant

This theme captured findings relating to the immediate period after the loss, reflecting initial thoughts about subsequent pregnancies and the point at which pregnancy planning emerges as an idea.

Immediacy of thoughts

Eight studies reported data suggesting that parents form a clear idea about whether or not they wish to try to conceive again soon after the death of their baby[19, 21, 43-45, 47, 50, 51].

"Absolutely immediately…. Whilst we were waiting to be induced, we were talking about next time."[21]

The decision for a subsequent pregnancy may be influenced and made more difficult by a range of inter-related variables[16, 43, 47, 51]. These included whether there were existing children, the number of losses, age, experiences of pregnancy, relationship status and whether the pregnancy was planned. Initial decisions sometimes changed over time[51].

Differences in reactions between partners

Six studies considered the experiences of both parents; four found that parents sometimes disagreed on the decision to try for another baby[16, 19, 49, 51]. This was a key observation one study, where fathers were observed to be more reluctant to consider a future pregnancy[19].

"My husband was saying he didn't want any more children. So I don't know if you count this much but someone who has lost a baby, for me I would have been pregnant coming out of the hospital again I wanted to be pregnant again that badly and then he was saying he didn't want any more at all and he wouldn't discuss it until after we got the results from the hospital."[19]

In three studies[19, 47, 49], men were reported to carry a different burden to women, in particular taking on the role of 'protector' who needed to be emotionally strong[19, 47, 49], and less likely to express their own fears [19, 47, 49].

For peer review only - http://bmjopen.bmj.com/site/about/guidelines.xhtml

Theme two: personal reactions

Unique experiences and reactions to loss influenced how each individual engaged with the concept of future pregnancy decisions.

Emotional response

Emotional responses to the death of a baby are unique to each parent and may influence, but also shape, understandings of the self and identity. In turn, these impact decisions about becoming pregnant after loss. In four studies, participants described the death as a void that could only be filled by a subsequent pregnancy[15, 21, 43, 50].

"Emotionally everything had been geared towards having a baby and then there was a big hole, a baby-shaped hole which was much bigger than a baby."[21]

Aspirations for a family

Findings from eleven studies highlighted how pregnancy and childbirth are conceptualised within individuals' life narratives, and how parents often have pre-existing expectations about their reproductive aspirations. Experiencing a perinatal death prompts parents to reconsider their life goals[19, 45].

"…but I was 29 when she was born, and I had this vision in my head that I was going to have all my kids by the time I was 30, and that wasn't working out, and so we better get on…."[16]

Easing perinatal grief

Five studies[21, 43, 45, 50, 51] reported that, for some parents, a subsequent pregnancy was considered essential for their recovery, eased feelings of perinatal grief, and gave hope for the future.

"I knew it was going to be a long process and it helped me recover because it made me focus on the future and I was more hopeful." [51]

However, not all parents were so certain that a subsequent pregnancy would aid recovery, and needed longer to overcome the grief and feel ready[19, 21, 43, 51]. These framings set the scene for

For peer review only - http://bmjopen.bmj.com/site/about/guidelines.xhtml

future pregnancy planning, which emerged and developed with reference to how an individual has interpreted the meaning of the prior loss in relation to their sense of self.

Theme three: social network influences

Future pregnancy planning decisions could be influenced by an individual's social network and the cultural norms and expectations within their social group. Comments and assumptions by others about future planning could be interpreted as supportive or distressing, depending on the context.

Altered relationships and social networks

Eight studies[16, 19, 44, 46, 48, 50, 52, 53] highlighted how relationships and social networks changed after a perinatal death. In three studies, some parents reported much support thereby reinforcing bonds with their social network[21, 48, 52]. However, in five studies, parents felt that friends and family were not supportive, and relationships were profoundly altered following unhelpful attitudes and responses[19, 44, 46, 48, 52]. As a result, bereaved parents may withdraw or isolate themselves from friends, family and colleagues to avoid hurt[19, 44, 46, 48, 52], and avoid discussing their loss to prevent their friends and family feeling uncomfortable[52].

"Everything is okay with my friends, as long as I don't talk about my baby. When I do, they look away and change the subject. What am I supposed to do? I need to talk!"[52]

Cultural norms and expectations

Nine studies discussed the impact of cultural norms on decisions about subsequent pregnancy[16, 19, 44-48, 50, 52]. Parents in four studies noted other people's discomfort, reporting that such uneasiness led to unsupportive reactions[16, 21, 46, 52].

In some cultures, while there are strong expectations for women to bear children, stillbirth is regarded as taboo and parents are not expected to see or to discuss their dead baby [44, 45]. A striking finding was the guilt and sense of failure that was reported following a perinatal death in this environment[44, 45]. This led some women to pursue pregnancy as soon as possible to undo the sense of wrong doing associated with stillbirth[44] and fulfil the cultural expectations of being a successful mother[45].

For peer review only - http://bmjopen.bmj.com/site/about/guidelines.xhtml

"I am trying to have a baby again. As long as I can have a baby, my mother-in-law can't look down on me. Also, failing to deliver a baby makes me feel that I am not a woman. Every woman should be able to deliver a baby – that's what makes a woman so different from a man."[44]

Theme four: planning or timing subsequent pregnancy

Eleven studies reported discussion of planning or timing a subsequent pregnancy[16, 19, 21, 43, 46, 48-53]. In six studies, parents felt that the timing of a subsequent pregnancy should be based on personal reasons and individual experience, rather than the thoughts of others, including medical professionals[19, 21, 43, 46, 51, 52]. Women spoke of listening to their body and trusting their own feelings[21, 43]. Planning a subsequent pregnancy often involved overcoming barriers[16, 43, 50, 51, 53], and sometimes conflicting messages from health professionals[52].

Barriers – practical and emotional

In five studies, parents cited a range of emotional and medical challenges as barriers to conceiving after loss[16, 43, 50, 51, 53]. Parents faced a period of ambiguity and uncertainty when planning their next pregnancy[16, 19, 21, 48, 50, 51, 53], and a loss of control associated with a long conception period[48]. Such uncertainty was intensified by practical factors, such as the number of previous perinatal deaths experienced, advanced maternal age, financial strain, parent relationship status and fertility problems[48, 51, 53]. High levels of anxiety and fear were linked to the lack of reassurance that a subsequent pregnancy would end successfully[16, 19, 46, 49, 50, 53].

"The last time I was actually very impatient because after that last miscarriage it lasted a year and a half before we were pregnant again. So I thought it would take a year and a half again to become pregnant so we tried again a month after the miscarriage." [48]

Such barriers may redefine decisions about how many children parents wish to have[53], or how soon parents try to conceive, with some deciding to delay to make sure they felt emotionally and physically ready for pregnancy[21, 43, 48, 50, 51].

Health professional advice

Seven studies discussed the involvement of health professionals in the inter-pregnancy period. This included answering medical questions, providing information and advice about

For peer review only - http://bmjopen.bmj.com/site/about/guidelines.xhtml

subsequent pregnancy and reassurance about the level of care that would be received for a subsequent pregnancy[16, 19, 43, 48, 49, 51, 52].

One early study focused specifically on mothers' perceptions of medical advice about timing of a subsequent pregnancy[43]. Five women were advised to wait less than six months before trying to conceive, matching the women's expectations, as they felt an urgent need to get pregnant[43]. Fourteen were advised to wait at least six months, 11 of whom found this advice unacceptable mainly due to the strong desire to have a baby:

"[12 months] is an eternity....I had all this parenting energy and nowhere to direct it...." [43]

Five women in this study received no specific advice about waiting. These women appreciated this both at the time and in hindsight, as it empowered them to make their own informed decision based on their individual needs. This was a consistent finding in other studies[21, 43, 51, 52].

Health professional information and reassurance

Concerned parents sought reassurance and information from health professionals, for example, about the risks of another negative outcome and the type of specialised care and emotional support they might expect to receive in a future pregnancy[16, 19, 21, 43, 46, 50-52].

"...they basically told us that we had the same chance of it happening to us again as if it had never happened to us. The same as any couple walking down the street. But the only thing I kept saying was that it did happen to us you know so they can't give any reassurances."[19]

However, not all parents received such advice and support[21], and although parents can be informed of level of risk, it was not possible to guarantee that there will not be another negative outcome in a future pregnancy[19].

Discussion

Many parents decide whether or not they wanted to have another pregnancy shortly after the loss. However, parents sometimes disagreed with their partners and early decisions could evolve over time. A key theme was the range of unique and personal reactions to loss. These experiences were intrinsically linked to decisions surrounding the timing of subsequent

For peer review only - http://bmjopen.bmj.com/site/about/guidelines.xhtml

pregnancy, which in turn influenced how participants engaged with future pregnancy decision-making. Future pregnancy planning may be influenced by an individual's social network and the cultural norms that guide people within their social group. The taboo that surrounds perinatal death makes it a difficult subject to discuss, potentially leaving bereaved parents feeling isolated and lonely. A salient finding was the assertion that parents should be provided with information about timing subsequent pregnancy, rather than prescriptive advice or specific recommendations[21, 43, 51, 52].

This review analysed and synthesised qualitative data from primary studies describing the experiences of bereaved parents in their transition from perinatal death to pregnancy, an area largely overlooked in the literature to date. The sample included a range of experiences from both parents, across eight countries, and included parents who had experienced different types of fetal or perinatal death. The themes identified were supported by evidence across all 15 studies. We used standardised and comprehensive methods, with explicit criteria, double blind screening, quality rating and data extraction, thus reducing the risk of bias.

Resource constraints limited the review to journal articles published in English. Relevant material was identified in other sources including abstracts and theses, and some themes may not have been identified. The majority of study participants were mothers; fathers' experiences may be underrepresented. All studies were undertaken in high income countries, and participants were mainly educated and living with the father of the baby. This has implications for the wider applicability of the findings.

There are inherent limitations associated with systematically reviewing qualitative studies, since only data which has been selected for presentation within the study is available, rather than the totality of the data collected. The quality of a systematic review further depends on the quality of the studies it includes. While the majority of the included studies were rated as good quality, and care was taken to ensure that themes were represented across multiple studies, one study rated as average quality contributed to six of the ten subthemes and dominated the findings in the subtheme regarding health professional advice. Thus, caution should be exercised regarding the wider applicability of this theme.

Investigating subsequent pregnancy decisions was the main aim of five studies[19, 21, 43, 48, 51], while ten studies reported relevant findings from studies where this was not the main focus.

For peer review only - http://bmjopen.bmj.com/site/about/guidelines.xhtml

The findings suggested that some parents develop a clear idea soon after perinatal death whether or not they wish to pursue a subsequent pregnancy. This reaction to pregnancy loss may reflect a strong desire to leave the liminal phase that parents experience following the death of a baby, whereby the nebulous identity of becoming non-pregnant leaves parents stranded between the stable states of being pregnant and parenthood[52, 54]. Other explanations include the theory that mothers and fathers assume their role of parents early in pregnancy[55], with psychological preparation for parenthood beginning before conception[56]. Jaffe and Diamond further suggest that people determine their own 'reproductive stories' as early as their own childhood; the perinatal death leaves this desire unfulfilled until a living child is born[57]. Whilst there is insufficient data to conclude that all parents decided quickly about subsequent pregnancy, we suggest that some parents may welcome opportunities to discuss their plans earlier than health professionals may assume. Conway and Russell emphasise the need to understand the most appropriate type and timing of health professional intervention after loss[49].

The data described a range of reactions to loss, and highlight the personal and individual nature of timing a subsequent pregnancy. This is consistent with other studies[18, 58-63 64 60]. Our findings suggest that parents may feel increased isolation after fetal or perinatal death, due to the taboo nature of the topic [18, 65, 66]. Social withdrawal has also been identified as an expression of grief following bereavement[67, 68].

The needs of bereaved parents during the period when they are contemplating becoming pregnant again has received substantially less interest than the need for additional support during subsequent pregnancy. Several studies have explored the experiences of parents during pregnancy following loss, and services have been developed to respond to their need for increased support. The process of conceiving the subsequent pregnancy is not discussed in these reports[22, 28, 69-71]. It is possible that health professionals do not consider involvement in such decisions as part of their role. However, there are a number of causes or risk factors for fetal and perinatal death which may be amenable to intervention in the inter-pregnancy interval, for example diabetes, obesity or smoking. This review highlights that the window of opportunity to influence these factors and support parents in reducing the risks in the next pregnancy may be relatively short. Health professionals may play an important role as a bridge between medical information and emotional support in the inter-pregnancy interval.

For peer review only - http://bmjopen.bmj.com/site/about/guidelines.xhtml

Conclusion

Many parents think about becoming pregnant again very soon after experiencing a pregnancy loss, and health professionals should anticipate the need to facilitate conversations from the very earliest point. Providing personalised and flexible support may be challenging for health professionals working within healthcare systems which may not easily adapt to differing needs. Further research should address the need to better understand the decision-making process, support requirements and challenges that parents face in the inter-pregnancy interval following pregnancy loss, and address how bereavement services can integrate better with pregnancy preparation services. This could be of particular benefit to those with medical conditions, such as pre-existing diabetes, where effective preparation for pregnancy and delayed conception have been shown to significantly improve outcomes [72, 73].

3467

Disclosure of interests All authors confirm that they have no interests to declare.

Contribution to authorship ED contributed to protocol development, performed the searches, reviewed the papers, conducted the thematic synthesis and wrote the first draft of the article. RB developed the idea for the study, contributed to protocol development, reviewed papers for inclusion, quality assessment and data extraction, and contributed to writing the final article. RG developed the idea for the study, contributed to protocol development, reviewed papers for inclusion, quality assessment and data extraction, supervised and reviewed the thematic synthesis and contributed to writing the final article. JR developed the idea for the study, contributed to protocol development, reviewed papers for inclusion, quality assessment and data extraction, and contributed to writing the final article

Details of ethics approval No ethical approval required.

Patient involvement Patients' and participants views were not sought.

PROSPERO registration: CRD42018112839

Funding: This work was supported by the Economic and Social Research Council [grant number ES/P000762/1].

Data availability: All data used in the analysis is available in the published papers. Additional data available from the authors comprises the text extracted from the papers relevant to the review question and used in the thematic analysis.

For peer review only - http://bmjopen.bmj.com/site/about/guidelines.xhtml

Figure 1: Themes and subthemes

For peer review only

For peer review only - http://bmjopen.bmj.com/site/about/guidelines.xhtml

References

1. World Health Organization. Neonatal and perinatal mortality: country, regional and global estimates. Ahmann E, Zupan J, World Health O, editors. Geneva: World Health Organization; 2006.
2. Fretts RC. Etiology and prevention of stillbirth. American Journal of Obstetrics and Gynecology. 2005;193(6):1923-35.
3. Office for National Statistics. Childhood, Infant and Perinatal Mortality in England and Wales 2015: ONS; 2015 [Available from: https://www.ons.gov.uk/peoplepopulationandcommunity/birthsdeathsandmarriages/deaths/bulletins/childhoodinfantandperinatalmortalityinenglandandwales/2015.
4. Statham H, Green JM. The effects of miscarriage and other "unsuccessful" pregnancies on feelings early in a subsequent pregnancy. Journal of Reproductive and Infant Psychology. 1994;12(1):45-54.
5. Woods JR, Woods JLE. Loss during pregnancy or in the newborn period. Pitman, New Jersey: Jannetti Publications; 1997.
6. Love ER, Bhattacharya S, Smith NC, Bhattacharya S. Effect of interpregnancy interval on outcomes of pregnancy after miscarriage: retrospective analysis of hospital episode statistics in Scotland. BMJ. 2010;341:c3967.
7. Fockler ME, Ladhani NNN, Watson J, Barrett JFR. Pregnancy subsequent to stillbirth: Medical and psychosocial aspects of care. Seminars in Fetal and Neonatal Medicine. 2017;22(3):186-92.
8. Gold KJ, Leon I, Chames MC. National survey of obstetrician attitudes about timing the subsequent pregnancy after perinatal death. American Journal of Obstetrics and Gynecology. 2010;202(4):357.
9. Kangatharan C, Labram S, Bhattacharya S. Interpregnancy interval following miscarriage and adverse pregnancy outcomes: systematic review and meta-analysis. Human Reproduction Update. 2017;23(2):221-31.
10. World Health Organization. Report of a WHO Technical Consultation on Birth Spacing. Geneva 2005.
11. Conde-Agudelo A, Rosas-Bermudez A, Castaño F, Norton MH. Effects of Birth Spacing on Maternal, Perinatal, Infant, and Child Health: A Systematic Review of Causal Mechanisms. Studies in Family Planning. 2012;43(2):93-114.

For peer review only - http://bmjopen.bmj.com/site/about/guidelines.xhtml

12. Buchmayer SM, Sparén P, Cnattingius S. Previous pregnancy loss: Risks related to severity of preterm delivery. American Journal of Obstetrics and Gynecology. 2004;191(4):1225-31.
13. Bhattacharya S, Townend J, Shetty A, Campbell D, Bhattacharya S. Does miscarriage in an initial pregnancy lead to adverse obstetric and perinatal outcomes in the next continuing pregnancy? BJOG: An International Journal of Obstetrics & Gynaecology. 2008;115(13):1623-9.
14. Hughes PM, Turton P, Evans CD. Stillbirth as risk factor for depression and anxiety in the subsequent pregnancy: cohort study. BMJ. 1999;318(7200):1721-4.
15. Grout LA, Romanoff BD. The myth of the replacement child: parents' stories and practices after perinatal death. Death Studies. 2000;24(2):93-113.
16. Phipps S. The subsequent pregnancy after stillbirth: Anticipatory parenthood in the face of uncertainty. The International Journal of Psychiatry in Medicine. 1986;15(3):243-64.
17. Goldstein RR, Croughan MS, Robertson PA. Neonatal outcomes in immediate versus delayed conceptions after spontaneous abortion: a retrospective case series. American Journal of Obstetrics and Gynecology. 2002;186(6):1230-4.
18. Burden C, Bradley S, Storey C, Ellis A, Heazell AE, Downe S, et al. From grief, guilt pain and stigma to hope and pride - a systematic review and meta-analysis of mixed-method research of the psychosocial impact of stillbirth. BMC Pregnancy & Childbirth. 2016;16(9):1-12.
19. Meaney S, Everard CM, Gallagher S, O'Donoghue K. Parents' concerns about future pregnancy after stillbirth: a qualitative study. Health Expectations. 2017;20(4):555-62.
20. Forrest GC, Standish E, Baum JD. Support after perinatal death: a study of support and counselling after perinatal bereavement. BMJ. 1982;285(6353):1475-9.
21. Lee L, McKenzie-McHarg K, Horsch A. Women's Decision Making and Experience of Subsequent Pregnancy Following Stillbirth. Journal of Midwifery & Women's Health. 2013;58(4):431-9.
22. DeBackere KJ, Hill PD, Kavanaugh KL. The Parental Experience of Pregnancy After Perinatal Loss. Journal of Obstetric, Gynecologic, & Neonatal Nursing. 2008;37(5):525-37.
23. Mills TA, Ricklesford C, Cooke A, Heazell AE, Whitworth M, Lavender T. Parents' experiences and expectations of care in pregnancy after stillbirth or neonatal death: a metasynthesis. BJOG: An International Journal of Obstetrics & Gynaecology. 2014;121(8):943-50.

For peer review only

24. Cuisinier M, Janssen H, De Graauw C, Bakker S, Hoogduin C. Pregnancy following miscarriage: course of grief and some determining factors. Journal of Psychosomatic Obstetrics & Gynecology. 1996;17(3):168-74.
25. August EM, Salihu HM, Weldeselasse H, Biroscak BJ, Mbah AK, Alio AP. Infant mortality and subsequent risk of stillbirth: a retrospective cohort study. Bjog. 2011;118(13):1636-45.
26. Monari F, Facchinetti F. Management of subsequent pregnancy after antepartum stillbirth. A review. The Journal of Maternal-Fetal & Neonatal Medicine. 2010;23(10):1073-84.
27. Robson SJ, Leader LR. Management of subsequent pregnancy after an unexplained stillbirth. Journal of Perinatology. 2010;30:305-10.
28. Lamb EH. The Impact of Previous Perinatal Loss on Subsequent Pregnancy and Parenting. The Journal of Perinatal Education. 2002;11(2):33-40.
29. Robson SJ, Leader LR, Dear KBG, Bennett MJ. Women's expectations of management in their next pregnancy after an unexplained stillbirth: An Internet-based empirical study. Australian and New Zealand Journal of Obstetrics and Gynaecology. 2009;49(6):642-6.
30. Cooke A, Smith D, Booth A. Beyond PICO:The SPIDER Tool for Qualitative Evidence Synthesis. Qualitative Health Research. 2012;22(10):1435-43.
31. Stern C, Jordan, Z., McArthur, A. Developing the Review Question and Inclusion Criteria. AJN: The American Journal of Nursing. 2014;114(4):53-6.
32. Methley AM, Campbell S, Chew-Graham C, McNally R, Cheraghi-Sohi S. PICO, PICOS and SPIDER: a comparison study of specificity and sensitivity in three search tools for qualitative systematic reviews. BMC Health Services Research. 2014;14(1):579-89.
33. Ouzzani M, Hammady H, Fedorowicz Z, Elmagarmid A. Rayyan — a web and mobile app for systematic reviews. Systematic Reviews. 2016;5(210).
34. Dixon-Woods M, Bonas S, Booth A, Jones DR, Miller T, Sutton AJ, et al. How can systematic reviews incorporate qualitative research? A critical perspective. Qualitative Research 2006;6(1):27-44.
35. Evans D. Database searches for qualitative research. Journal of the Medical Library Association. 2002;90(3):290-3.
36. Jones ML. Application of systematic review methods to qualitative research: practical issues. Journal of Advanced Nursing. 2004;48(3):271-8.
37. Dixon-Woods M, Sutton A, Shaw R, Miller T, Smith J, Young B, et al. Appraising qualitative research for inclusion in systematic reviews: a quantitative and qualitative comparison of three methods. Journal of Health Services Research & Policy. 2007;12(1):42-7.

For peer review only - http://bmjopen.bmj.com/site/about/guidelines.xhtml

38. Butler A, Hall H, Copnell B. A guide to writing a qualitative systematic review protocol to enhance evidence-based practice in nursing and health care. Worldviews on Evidence-Based Nursing. 2016;13(3):241-9.

39. McKibbon KA, Wilczynski NL, Haynes RB. Developing Optimal Search Strategies for Retrieving Qualitative Studies in PsycINFO. Evaluation & the Health Professions. 2006;29(4):440-54.

40. Lockwood C, Porrit K, Munn Z, Rittmeyer L, Salmond S, Bjerrum, M, Loveday H, Carrier J, Stannard, D. Systematic reviews of qualitative evidence. In: Aromataris EM, Z. , editor. Joanna Briggs Institute Reviewer's Manual: The Joanna Briggs Institute; 2017.

41. Critical Appraisal Skills Programme (CASP). Qualitative Checklist 2018 [Available from: https://casp-uk.net/wp-content/uploads/2018/01/CASP-Qualitative-Checklist.pdf.

42. Harden A, Thomas J. Methodological issues in combining diverse study types in systematic reviews. International Journal of Social Research Methodology. 2005;8(3):257-71.

43. Davis DL, Stewart M, Harmon RJ. Postponing Pregnancy after Perinatal Death: Perspectives on Doctor Advice. Journal of the American Academy of Child & Adolescent Psychiatry. 1989;28(4):481-7.

44. Hsu M, Tseng Y, Kuo L. Transforming loss: Taiwanese women's adaptation to stillbirth. Journal of Advanced Nursing. 2002;40(4):387-95.

45. Tseng YF, Chen CH, Wang HH. Taiwanese women's process of recovery from stillbirth: a qualitative descriptive study. Research in Nursing & Health. 2014;37(3):219-28.

46. Bansen SS, Stevens HA. Women's experiences of miscarriage in early pregnancy. Journal of Nurse-Midwifery. 1992;37(2):84-90.

47. Cecil R. "I wouldn't have minded a wee one running about": Miscarriage and the family. Social Science & Medicine. 1994;38(10):1415-22.

48. Ockhuijsen HDL, van den Hoogen A, Boivin J, Macklon NS, de Boer F. Pregnancy After Miscarriage: Balancing Between Loss of Control and Searching for Control. Research in Nursing & Health. 2014;37(4):267-75.

49. Conway K, Russell G. Couples' grief and experience of support in the aftermath of miscarriage. British Journal of Medical Psychology. 2000;73(4):531-45.

50. Carlsson T, Bergman G, Karlsson A-M, Wadensten B, Mattsson E. Experiences of termination of pregnancy for a fetal anomaly: A qualitative study of virtual community messages. Midwifery. 2016;41:54-60.

For peer review only

For peer review only - http://bmjopen.bmj.com/site/about/guidelines.xhtml

51. Keim MC, Fortney CA, Shultz EL, Winning A, Gerhardt CA, Baughcum A. Parent Distress and the Decision to Have Another Child After an Infant's Death in the NICU. JOGNN: Journal of Obstetric, Gynecologic & Neonatal Nursing. 2017;46(3):446-55.

52. de Montigny F, Dumas L, Beaudet L. A baby has died: The impact of perinatal loss on family social networks. Journal of Obstetric, Gynecologic & Neonatal Nursing. 1999;28(2):151-6.

53. Cacciatore J, DeFrain J, Jones KLC. When a Baby Dies: Ambiguity and Stillbirth. Marriage & Family Review. 2008;44(4):439-54.

54. Reiheld A. "The Event That Was Nothing": Miscarriage as a Liminal Event. Journal of Social Philosophy. 2015;46(1):9-26.

55. Rubin R. Attainment of the maternal role: Part 1. Processes. Nursing Research. 1967;16(3):237-45.

56. Shapiro CH. Infertility and pregnancy loss: A guide for helping professionals. 1988;251.

57. Jaffe J, Diamond MO. When the reproductive story goes awry: Trauma and loss. Reproductive Trauma : Psychotherapy with Infertility and Pregnancy Loss Clients. Washington DC, US: The American Psychology Association; 2011.

58. Brooten D, Youngblut JM, Hannan J, Caicedo C, Roche R, Malkawi F. Infant and child deaths: Parent concerns about subsequent pregnancies. Journal of the American Association of Nurse Practitioners. 2015;27(12):690-7.

59. Koopmans L, Wilson T, Cacciatore J, Flenady V. Support for mothers, fathers and families after perinatal death. Cochrane Database of Systematic Reviews. 2013(6).

60. Ellis A, Chebsey C, Storey C, Bradley S, Jackson S, Flenady V, et al. Systematic review to understand and improve care after stillbirth: a review of parents' and healthcare professionals' experiences. BMC Pregnancy and Childbirth. 2016;16(1):16.

61. Erlandsson K, Lindgren H, Malm MC, Davidsson-Bremborg A, Rådestad I. Mothers' experiences of the time after the diagnosis of an intrauterine death until the induction of the delivery: a qualitative Internet-based study. The journal of obstetrics and gynaecology research. 2011;37(11):1677-84.

62. Trulsson O, Rådestad I. The silent child - mothers' experiences before, during, and after stillbirth. Birth: Issues in Perinatal Care. 2004;31(3):189-95.

63. Malacrida CA. Perinatal death: helping parents find their way. Journal of Family Nursing. 1997;3(2):130-48.

64. Turton P, Badenhorst W, Hughes P, Ward J, Riches S, White S. Psychological impact of stillbirth on fathers in the subsequent pregnancy and puerperium. The British Journal of Psychiatry. 2006;188:165-72.

For peer review only - http://bmjopen.bmj.com/site/about/guidelines.xhtml

65. Hutti MH. An exploratory study of the miscarriage experience. Health Care for Women International. 1986;7(5):371-89.

66. Swanson-Kauffman KM. Caring in the instance of unexpected early pregnancy loss. Topics in clinical nursing. 1986;8(2):37-46.

67. Asplin N, Wessel H, Marions L, Öhman SG. Pregnancy termination due to fetal anomaly: Women's reactions, satisfaction and experiences of care. Midwifery. 2014;30(6):620-7.

68. Capitulo KL. Evidence for healing interventions with perinatal bereavement. MCN: The American Journal of Maternal/Child Nursing. 2005;30(6):389-96.

69. Côté-Arsenault D, Marshall R. One Foot In-One Foot Out: Weathering the Storm of Pregnancy After Perinatal Loss. Research in Nursing & Health. 2000;23(6):473-85.

70. Hunter A, Tussis, L., MacBeth, A. The presence of anxiety, depression and stress in women and their partners during pregnancies following perinatal loss: A meta-analysis. Journal of Affective Disorders. 2017;223(Supplement C):153-64.

71. Mills TA, Ricklesford C, Heazell AEP, Cooke A, Lavender T. Marvellous to mediocre: findings of national survey of UK practice and provision of care in pregnancies after stillbirth or neonatal death. BMC Pregnancy and Childbirth. 2016;16(101).

72. McElvy SS, Miodovnik M, Rosenn B, Khoury JC, Siddiqi T, Dignan PSJ, et al. A focused preconceptional and early pregnancy program in women with type 1 diabetes reduces perinatal mortality and malformation rates to general population levels. Journal of Maternal-Fetal Medicine. 2000;9(1):14-20.

73. Murphy HR, Roland JM, Skinner TC, Simmons D, Gurnell E, Morrish NJ, et al. Effectiveness of a regional prepregnancy care program in women with type 1 and type 2 diabetes: benefits beyond glycemic control. Diabetes Care. 2010;33(12):2514-20.

For peer review only

For peer review only - http://bmjopen.bmj.com/site/about/guidelines.xhtml

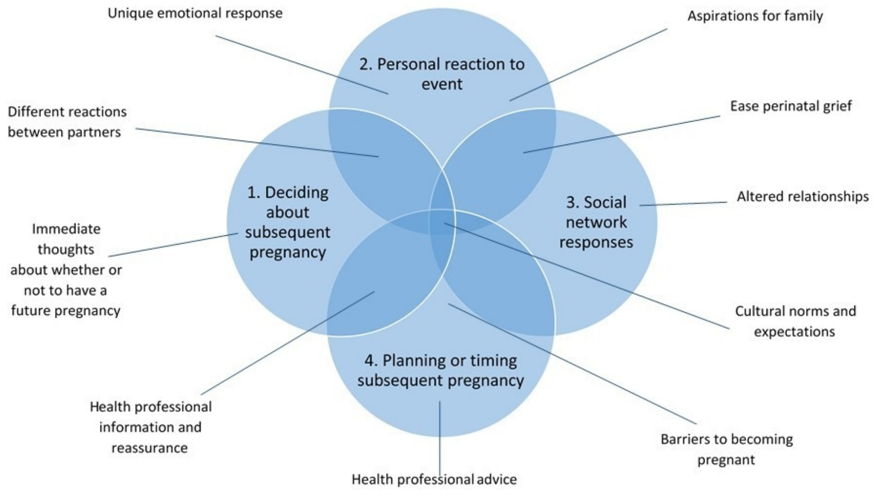

86x44mm (300 x 300 DPI)

Appendix S1

	Search Terms
1.	(Stillbirth or still-birth or "perinatal loss" or "perinatal death" or "perinatal mortality" or "neonatal death" or miscarriage or "reproductive loss" or "pregnancy complications" or "fetal death" or "fetal resorption" or "foetal death" or "foetal resorption").mp.
2.	(Infant Mortality or Pregnancy outcome or Fetal Diseases or Foetal Diseases or Pregnancy Complications).mp.
3.	(baby death or newborn death or new-born death).mp.
4.	(abortion, spontaneous or abortion or "termination of pregnancy").mp. [mp=title, abstract, original title, name of substance word, subject heading word, keyword heading word, protocol supplementary concept word, rare disease supplementary concept word, unique identifier, synonyms]
5.	(behavior or behaviour or emotion* or psychosocial or "emotional state" or "psychological state").mp. [mp=title, abstract, original title, name of substance word, subject heading word, keyword heading word, protocol supplementary concept word, rare disease supplementary concept word, unique identifier, synonyms]
6.	(bereavement or grief or expressed emotion).mp.
7.	(experience* or perception* or feeling* or attitude* or voice*).mp.
8.	(pregnancy or pre-pregnancy or "subsequent pregnancy" or "previous pregnancy" or "past pregnancy").mp.
9.	(pre-conception or preconception or "preconception care" or "prenatal care").mp.
10.	(peri-conception or periconception).mp.
11.	(interpregnancy or inter-pregnancy or "interpregnancy interval" or "inter-pregnancy interval").mp.
12.	("Birth Interval*" or "birth spacing").mp. [mp=title, abstract, original title, name of substance word, subject heading word, keyword heading word, protocol supplementary concept word, rare disease supplementary concept word, unique identifier, synonyms]
13.	(contraception or "contraception behavio*" or interconception or inter-conception or postpartum or post-partum).mp.
14.	(((Decision/ adj4 pregnanc*.mp.) or plan*.mp.) adj3 pregnancy.mp.) or "family planning".mp. [mp=title, abstract, original title, name of substance word, subject heading word, keyword heading word, protocol supplementary concept word, rare disease supplementary concept word, unique identifier, synonyms]
15.	(parent* or father or mother* or wom*n or "single parent" or spouse* or relationship* or "partner relationship*").mp. [mp=title, abstract, original title, name of substance word, subject heading word, keyword heading word, protocol supplementary concept word, rare disease supplementary concept word, unique identifier, synonyms]
16.	(psychosocial or psychological state).mp.

For peer review only

17.	(mixed-method or multi-method).mp. or empirical research/ or grounded theory/ or qualitative research/ or hermeneutics/ or interdisciplinary research/ or phenomenological research.mp. or phenomenology.mp. [mp=title, abstract, original title, name of substance word, subject heading word, keyword heading word, protocol supplementary concept word, rare disease supplementary concept word, unique identifier, synonyms]
18.	(interview or focus group or survey or questionnaire or diar* or cohort stud* or case stud* or ethnograph* or retrospective or longitudinal or observation*).mp. [mp=title, abstract, original title, name of substance word, subject heading word, keyword heading word, protocol supplementary concept word, rare disease supplementary concept word, unique identifier, synonyms]
19.	17 or 18
20.	1 or 2 or 3 or 4
21.	5 or 6 or 7 or 14 or 16
22.	8 or 9 or 10 or 11 or 12 or 13
23.	15 and 19 and 20 and 21 and 22
24.	limit 23 to (abstracts and english language and humans and "qualitative (maximizes sensitivity)")
25.	20
26.	limit 20 to abstracts
27.	15 and 19 and 21 and 22 and 26
28.	limit 27 to (english language and humans and "qualitative (maximizes specificity)")

For peer review only

Appendix S2

PRISMA flow chart of search strategy and outcomes.

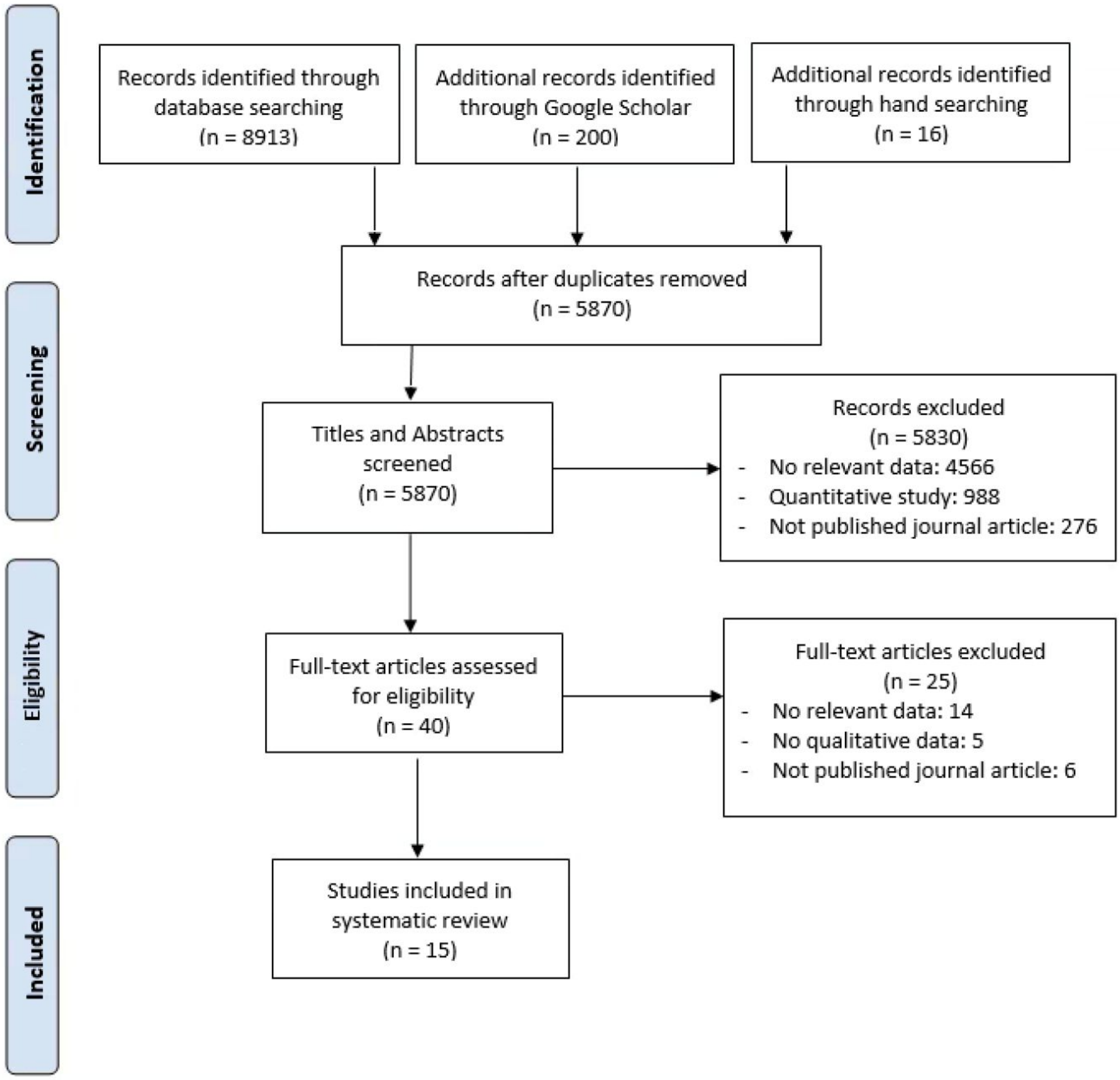

Table S1 – Summary of Included Studies

Author (Year)	*Title*	*Sampling size and method*	*Data collection method*	*Key characteristics of participants*	*Type of Loss*	*Location*
Bansen and Stevens (1992)[46]	Women's experiences of miscarriage in early pregnancy	*n=10* Convenience sample	Open-ended, guided interview	- Mothers interviewed 2-5 months after miscarrying first desired pregnancy<br>- Aged: 23-33 (mean= 28.5 years)<br>- Ethnicity: white	Early miscarriage 6-15 weeks gestation (mean= 8.8 weeks)	USA
Cacciatore et al., (2008)[53]	When a baby dies: Ambiguity and stillbirth.	*n=74* Convenience sample	Retrospective analysis of focus group data	- Grieving mothers, fathers, sisters, brothers, grandmothers, grandfathers and an aunt<br>- No demographic information	Stillbirth	USA
Carlsson et al., (2016)[50]	Experiences of termination of pregnancy for fetal anomaly: A qualitative study of virtual community messages	*n= 122* Purposive sample	Cross-sectional study of messages in 2 virtual communities	- Majority mothers (*n=112*)<br>- Experience of TOPFA in past year<br>- 106 (86.9%) had previous experience of TOPFA<br>- No demographic information	TOPFA after 15 weeks gestation	Sweden
Cecil (1994)[47]	"I wouldn't have minded a wee one running about"	*n= 27* Convenience sample	Semi-structured interviews with follow up interviews	- Mothers<br>- Aged 19-43<br>- Religion: Protestant (*n=13*), Catholic (*n=13*), No Religion (*n=1*)<br>- Limited demographic information	First trimester miscarriage	*UK*
Conway and Russell (2000)[49]	Couples' grief and experience of support in the aftermath of miscarriage	*n= 71* Purposive sample	Prospective study. Questionnaire (48% open-ended)	- Mothers (*n=39*) accompanied by partners (*n=32*)<br>- Experience of miscarriage in past 4 months<br>- Previous children (mothers *n=22;* partners *n*=12)<br>- Socio-economic status tended towards average<br>- Limited demographic information	Miscarriage 5-16 weeks gestation (mean = 9.5 weeks)	*Australia*

For peer review only - http://bmjopen.bmj.com/site/about/guidelines.xhtml

For peer review only

Author (Year)	*Title*	*Sampling size and method*	*Data collection method*	*Key characteristics of participants*	*Type of Loss*	*Location*
Davis et al., (1989)[43]	Postponing Pregnancy after Perinatal Death: Perspectives on Doctor Advice	*n=24* Convenience sample	Open-ended interview	- Mothers<br>- Experience of stillbirth in past 1-10 years<br>- Previous children (*n=24*)<br>- No demographic information	Stillbirth	*USA*
de Montigny et al., (1999)[52]	A baby has died: the impact of perinatal loss on family social networks	*n=20* Convenience sample	Self-administered questionnaire consisting of 23 open-ended questions	- Mothers (*n=14)* and fathers (*n=6)*<br>- Experience of perinatal death in past 6 months<br>- Experience of multiple losses: >1 perinatal death (*n=6);* 4 perinatal deaths (*n=2)*; 1 neonatal death (*n=1)*<br>- Ethnicity: white; French-speaking; middle class	Perinatal death	*Canada*
Grout and Romanoff (2000)[15]	The myth of the replacement child: parents' stories and practices after perinatal death	*n=10* Purposive sample	Open-ended interview	- Parents of 7 families (3 couples, 1 father, 3 mothers)<br>- Experience of perinatal death in past 2-10 years<br>- Participants had a subsequent child between 3 and 5 years of age<br>- Experience of multiple losses (*n=3*)<br>- Previous children (*n=4*)<br>- Intact marriages<br>- Middle class; ethnicity: white	Stillbirth (including late-term miscarriage, or early infant death)	*USA*

For peer review only - http://bmjopen.bmj.com/site/about/guidelines.xhtml

Author (Year)	*Title*	*Sampling size and method*	*Data collection method*	*Key characteristics of participants*	*Type of Loss*	*Location*
Hsu et al., (2002)[44]	Transforming loss: Taiwanese women's adaption to stillbirth	n= 20<br>Purposive sample	Open-ended interview	- Mothers<br>- Experience of stillbirth in past year<br>- Aged: 22-40 (mean = 30 years)<br>- First pregnancy (*n=6*)<br>- Previous children (*n=7*)<br>- Ethnicity: Taiwanese<br>- Limited demographic information	Stillbirth	*Taiwan*
Keim et al., (2017)[51]	Parent distress and the decision to have another child after an infant's death in the Neonatal Intensive Care Unit (NICU)	*n=69*<br>Purposive sample	Semi-structured qualitative telephone interviews	- Mothers (*n=42*) and fathers (*n=27*) but technical difficulties meant interviews were analysed for mothers (*n=28*) and fathers (*n*=11)<br>- Experience of death in NICU in past 3 months-5 years<br>- Aged: 18 years +<br>- Approximately 2/3 had subsequent child<br>- Majority married; majority completed secondary education<br>- Ethnicity: majority white; English speaking	Death of neonatal infant in NICU	*USA*
Lee et al., (2013)[21]	Women's decision making and experience of subsequent pregnancy following stillbirth	*n=11*<br>Convenience sample	Interview	- Mothers<br>- Experience of stillbirth in past 6-12 months (mean=34 weeks)<br>- No mothers had given birth to subsequent child but some were pregnant (*n=8*) (9-25 weeks (mean = 14 weeks)<br>- Aged: 26-43 (mean = 32 years)<br>- Majority first pregnancy (*n=8*)<br>- Married or cohabiting; majority in full-time work	Stillbirth: 24+ weeks' gestation (mean = 36 weeks)	*UK*

For peer review only - http://bmjopen.bmj.com/site/about/guidelines.xhtml

Author (Year)	*Title*	*Sampling size and method*	*Data collection method*	*Key characteristics of participants*	*Type of Loss*	*Location*
				- Ethnicity: white British (*n=8*), Slovakian (*n=1*), Italian (*n=1*), white Asian (*n=1*); English-speaking		
Meaney et al., (2017)[19]	Parents' concerns about future pregnancy after stillbirth: a qualitative study	*n=15* Purposive sample	Semi-structured face-to-face interviews<br><br>Couples interviewed separately	- Mothers (*n=10*) and fathers (*n=5*)<br>- Experience of stillbirth in past 4-16 months<br>- Irish<br>- No demographic information	Stillbirth	*Ireland*
Ockhuijsen et al., (2014)[48]	Pregnancy after miscarriage: balancing between loss of control and searching for control	*n=24* Purposive sample	Semi-structured, face-to-face interviews	- Mothers<br>- Interviewed after 8 weeks of subsequent pregnancy. Those receiving fertility treatment were excluded<br>- Aged: 27-38 (mean=33 years)<br>- Prior experience of miscarriage 1-5 (median=2)<br>- Married or partnered; heterosexual<br>- Education ranged from secondary school to university<br>- Dutch speakers	Miscarriage	*Netherlands*
Phipps (1986)[16]	The subsequent pregnancy after stillbirth: anticipatory	*n=30* Purposive sample	Retrospective, self-reported,	- Couples<br>- Experience of stillbirth (*n=8) or* neonatal death (*n=7*)	Neonatal death (including stillbirth due to prematurity	*USA*

For peer review only - http://bmjopen.bmj.com/site/about/guidelines.xhtml

Author (Year)	*Title*	*Sampling size and method*	*Data collection method*	*Key characteristics of participants*	*Type of Loss*	*Location*
	parenthood in the face of uncertainty		open-ended interview<br><br>Couples interviewed together in all but one instance	- Subsequent child aged 5months – 3 years (mean = 15 months)<br>- Mother mean age: 30.2 years; Father mean age: 33.7 years<br>- Previous children (*n*=6)<br>- Homogenous, middle class sample<br>- Intact, two-parent families<br>- Ethnicity: Black (*n=1*), Hispanic (*n*=1), Caucasian (*n=13*)	or congenital anomalies)	
Tseng et al., (2014)[45]	Taiwanese women's process of recovery from stillbirth: A qualitative descriptive study	*n=21*<br>Purposive sample	38 in-depth interviews: face-to-face (*n=28*) and telephone (*n=4*)<br><br>11 participants took part in follow-up interviews	- Mothers<br>- Experienced stillbirth in past 6 months – 6 years (mean = 2.3 years)<br>- Aged: 23-43 (mean 31.9 years)<br>- Previous children (*n=9*)<br>- Married<br>- Employed (*n=14*)<br>- Ethnicity: Taiwanese<br>- Religion: Taiwanese folk beliefs (*n=8*), Buddhism (*n=4*), Taoism (*n=3*), Christianity (*n=2*), I-Kuan Tao (*n=2*)	Stillbirth (20-37 weeks gestation)	*Taiwan*

TOPFA – termination of pregnancy for fetal anomaly

For peer review only - http://bmjopen.bmj.com/site/about/guidelines.xhtml

Table S2: CASP Quality Assessment Results

CASP Question	Bansen and Stevens (1992)[46]	Cacciatore *et al.*, (2008)[53]	Carlsson *et al.*, (2016)[50]	Cecil (1994)[47]	Conway and Russell (2000)[49]	Davis *et al.*, (1989)[43]	de Montigny *et al.*, (1999)[52]	Grout and Romanoff (2000)[15]	Hsu *et al.*, (2002)[44]	Keim *et al.*, (2017)[51]	Lee *et al.*, (2013)[21]	Meaney *et al.*, (2017)[19]	Ockhuijsen *et al.*, (2014)[48]	Phipps (1986)[16]	Tseng *et al.*, (2014)[45]
1	Y	Y	Y	Y	Y	Y	Y	Y	Y	Y	Y	Y	Y	Y	Y
2	Y	Y	Y	Y	Y	Y	Y	Y	Y	Y	Y	Y	Y	Y	Y
3	Y	Y	Y	Y	Y	C	Y	Y	C	Y	Y	Y	Y	Y	Y
4	Y	Y	Y	Y	Y	C	Y	Y	C	Y	Y	C	Y	Y	Y
5	Y	C	Y	C	Y	C	Y	Y	C	Y	Y	Y	Y	Y	Y
6	Y	N	Y	N	N	N	N	C	Y	Y	C	N	N	C	N
7	N	N	Y	C	C	N	C	C	Y	Y	Y	Y	Y	N	Y
8	Y	N	Y	C	C	C	Y	C	C	Y	Y	Y	Y	C	Y
9	Y	N	Y	Y	Y	Y	Y	Y	Y	Y	Y	Y	Y	Y	Y
10	Y	Y	Y	N	Y	Y	Y	Y	Y	Y	Y	Y	Y	Y	Y
**CASP Score**	**18**	**11**	**20**	**13**	**16**	**12**	**17**	**17**	**16**	**20**	**19**	**17**	**18**	**16**	**18**
**Quality**	**Good**	**Average**	**Good**	**Average**	**Good**	**Average**	**Good**	**Good**	**Good**	**Good**	**Good**	**Good**	**Good**	**Good**	**Good**

Key:

For peer review only

Y – Yes = 2

C - Can’t Tell = 1

N- No = 0

For peer review only

Table S3 – Themes and subthemes emerging from the included studies

Author (year)	Deciding about becoming pregnant		Individual reaction to the event			Social network responses		Planning or timing subsequent pregnancy		
	Immediacy of thoughts	*Differences in reactions between parents*	*Emotional response to loss*	*Aspirations for a family*	*Ease perinatal grief*	*Altered relationships and social networks*	*Cultural norms and expectations*	*Barriers*	*Health professional advice*	*Health professional information and reassurance*
Bansen and Stevens			✓			✓	✓			✓
Cacciatore et al., (2008)[53]		✓	✓	✓		✓		✓		
Carlsson et al., (2016)[50]	✓		✓	✓	✓	✓	✓	✓		✓
Cecil (1994)[47]	✓	✓	✓				✓			
Conway and Russell (2000)[49]		✓	✓						✓	
Davis et al., (1989)[43]	✓		✓	✓	✓			✓	✓	✓
de Montigny et al., (1999)[52]			✓			✓	✓		✓	✓
Grout and Romanoff (2000)[15]			✓	✓						
Hsu et al., (2002)[44]	✓		✓	✓		✓	✓			
Keim et al., (2017)[51]	✓	✓	✓	✓	✓			✓	✓	✓
Lee et al., (2013)[21]	✓		✓	✓	✓					✓
Meaney et al., (2017)[19]	✓	✓	✓	✓		✓	✓		✓	✓
Ockhuijsen et al., (2014)[48]			✓	✓		✓	✓		✓	
Phipps (1986)[16]		✓	✓	✓		✓	✓	✓	✓	✓

Tseng et al., (2014)[45]	✓		✓	✓	✓		✓			

For peer review only

PRISMA 2009 Checklist

Section/topic	#	Checklist item	Reported on page #
**TITLE**			
Title	1	Identify the report as a systematic review, meta-analysis, or both.	1
**ABSTRACT**			
Structured summary	2	Provide a structured summary including, as applicable: background; objectives; data sources; study eligibility criteria, participants, and interventions; study appraisal and synthesis methods; results; limitations; conclusions and implications of key findings; systematic review registration number.	2
**INTRODUCTION**			
Rationale	3	Describe the rationale for the review in the context of what is already known.	3
Objectives	4	Provide an explicit statement of questions being addressed with reference to participants, interventions, comparisons, outcomes, and study design (PICOS).	3. SPIDER framework used in place of PICOS
**METHODS**			
Protocol and registration	5	Indicate if a review protocol exists, if and where it can be accessed (e.g., Web address), and, if available, provide registration information including registration number.	13
Eligibility criteria	6	Specify study characteristics (e.g., PICOS, length of follow-up) and report characteristics (e.g., years considered, language, publication status) used as criteria for eligibility, giving rationale.	3, 4
Information sources	7	Describe all information sources (e.g., databases with dates of coverage, contact with study authors to identify additional studies) in the search and date last searched.	4
Search	8	Present full electronic search strategy for at least one database, including any limits used, such that it could be repeated.	Appendix S1
Study selection	9	State the process for selecting studies (i.e., screening, eligibility, included in systematic review, and, if applicable, included in the meta-analysis).	4, table S1
Data collection process	10	Describe method of data extraction from reports (e.g., piloted forms, independently, in duplicate) and any processes for obtaining and confirming data from investigators.	4
Data items	11	List and define all variables for which data were sought (e.g., PICOS, funding sources) and any assumptions and simplifications made.	4

For peer review only

PRISMA 2009 Checklist

Risk of bias in individual studies	12	Describe methods used for assessing risk of bias of individual studies (including specification of whether this was done at the study or outcome level), and how this information is to be used in any data synthesis.	4, table S2
Summary measures	13	State the principal summary measures (e.g., risk ratio, difference in means).	n/a
Synthesis of results	14	Describe the methods of handling data and combining results of studies, if done, including measures of consistency (e.g., I^2) for each meta-analysis.	4, table S3

Section/topic	#	Checklist item	Reported on page #
Risk of bias across studies	15	Specify any assessment of risk of bias that may affect the cumulative evidence (e.g., publication bias, selective reporting within studies).	4, 11
Additional analyses	16	Describe methods of additional analyses (e.g., sensitivity or subgroup analyses, meta-regression), if done, indicating which were pre-specified.	n/a
**RESULTS**			
Study selection	17	Give numbers of studies screened, assessed for eligibility, and included in the review, with reasons for exclusions at each stage, ideally with a flow diagram.	Appendix S2
Study characteristics	18	For each study, present characteristics for which data were extracted (e.g., study size, PICOS, follow-up period) and provide the citations.	Table S1
Risk of bias within studies	19	Present data on risk of bias of each study and, if available, any outcome level assessment (see item 12).	Table S2
Results of individual studies	20	For all outcomes considered (benefits or harms), present, for each study: (a) simple summary data for each intervention group (b) effect estimates and confidence intervals, ideally with a forest plot.	n/a
Synthesis of results	21	Present results of each meta-analysis done, including confidence intervals and measures of consistency.	n/a
Risk of bias across studies	22	Present results of any assessment of risk of bias across studies (see Item 15).	n/a
Additional analysis	23	Give results of additional analyses, if done (e.g., sensitivity or subgroup analyses, meta-regression [see Item 16]).	n/a
**DISCUSSION**			
Summary of evidence	24	Summarize the main findings including the strength of evidence for each main outcome; consider their relevance to key groups (e.g., healthcare providers, users, and policy makers).	10
Limitations	25	Discuss limitations at study and outcome level (e.g., risk of bias), and at review-level (e.g., incomplete retrieval of identified research, reporting bias).	11
Conclusions	26	Provide a general interpretation of the results in the context of other evidence, and implications for future research.	12
**FUNDING**			

For peer review only - http://bmjopen.bmj.com/site/about/guidelines.xhtml

PRISMA 2009 Checklist

Funding	27	Describe sources of funding for the systematic review and other support (e.g., supply of data); role of funders for the systematic review.	13

From: Moher D, Liberati A, Tetzlaff J, Altman DG, The PRISMA Group (2009). Preferred Reporting Items for Systematic Reviews and Meta-Analyses: The PRISMA Statement. PLoS Med 6(7): e1000097. doi:10.1371/journal.pmed1000097

For more information, visit: **www.prisma-statement.org**.

For peer review only

For peer review only - http://bmjopen.bmj.com/site/about/guidelines.xhtml

BMJ Open

Pregnancy decisions after fetal or perinatal death: systematic review of qualitative research

Journal:	*BMJ Open*
Manuscript ID	bmjopen-2019-029930.R1
Article Type:	Original research
Date Submitted by the Author:	24-Oct-2019
Complete List of Authors:	Dyer, Eleanor; Newcastle University, Institute of Health & Society, Baddiley-Clark Building, Richardson Road, Newcastle, UK, NE2 4AX<br>Bell, Ruth; Newcastle University, Institute of Health & Society, Baddiley-Clark Building, Richardson Road, Newcastle, UK, NE2 4AX<br>Graham, Ruth; Newcastle University, School of Geography, Sociology and Politics<br>Rankin, Judith; Newcastle University, Institute of Health & Society, Baddiley-Clark Building, Richardson Road, Newcastle, UK, NE2 4AX
**Primary Subject Heading**:	Obstetrics and gynaecology
Secondary Subject Heading:	Reproductive medicine
Keywords:	MENTAL HEALTH, OBSTETRICS, PERINATOLOGY, QUALITATIVE RESEARCH

SCHOLARONE™
Manuscripts

Title: Pregnancy decisions after fetal or perinatal death: systematic review of qualitative research

Authors: Eleanor Dyer[1], Ruth Bell[1], Ruth Graham[2], Judith Rankin[1]

1. Institute of Health & Society
   Newcastle University
   Baddiley-Clark Building
   Richardson Road
   Newcastle upon Tyne

2. School of Geography, Politics and Sociology
   Newcastle University
   19 Windsor Terrace
   Newcastle upon Tyne
   NE1 7RU

Corresponding author: Professor Judith Rankin

Contact details:

Address:

Institute of Health & Society

Newcastle University

Baddiley-Clark Building

Richardson Road

Newcastle upon Tyne

NE2 4AX

Telephone number: +44 191 208 5267

Email: Judith.rankin@ncl.ac.uk

Running title: Pregnancy decisions after loss: qualitative systematic review

For peer review only

For peer review only - http://bmjopen.bmj.com/site/about/guidelines.xhtml

Abstract

Objectives To synthesise the findings of qualitative research exploring parents' experiences, views and decisions about becoming pregnant following a perinatal death or fetal loss.

Design: Systematic review and meta-synthesis of qualitative research.

Data Sources: Medline, Web of Science, CINAHL, PsycINFO, ASSIA, Embase, PUBMED, Scopus and Google Scholar.

Eligibility Criteria: Nine electronic databases were searched using predefined search terms. Articles published in English, in peer reviewed journals, using qualitative methods to explore the experiences and attitudes of bereaved parents following perinatal or fetal loss, were included.

Data Extraction and Synthesis: Qualitative data relating to first order and second order constructs were extracted and synthesised across studies using a thematic analysis.

Results: 15 studies were included. Four descriptive themes and 10 subthemes were identified. The descriptive themes were: deciding about subsequent pregnancy; diversity of reactions to the event; social network influences, and planning or timing of subsequent pregnancy. The decision to become pregnant after death is complex and varies between individuals and sometimes within couples. Decisions are often made quickly, in the immediate aftermath of a pregnancy loss, but may evolve over time. Bereaved parents may feel isolated from social networks.

Conclusions: There is an opportunity to support parents to prepare for a pregnancy after a fetal or perinatal loss, and conversations may be welcomed at an early stage. Health professionals may play an important role providing support lacking from usual social networks.

PROSPERO registration: CRD42018112839

Strengths and limitations of this study:

- This review synthesised qualitative data from primary studies describing the experiences of bereaved parents in their transition from perinatal death to pregnancy
- We used standardised methods, including double blind screening, quality rating and data extraction
- The themes identified were supported by evidence grounded in all included studies
- Study participants were mainly mothers from high income countries, with high levels of education and living with their partners, limiting the wider applicability of the findings

For peer review only - http://bmjopen.bmj.com/site/about/guidelines.xhtml

- The review was limited to peer-reviewed journal articles published in English

Introduction

Perinatal and fetal death remain common adverse pregnancy outcomes[1, 2], with a perinatal mortality rate of 6.6 per 1000 births in England and Wales[3], and estimated miscarriage rate of 25%-43%[4, 5]. Many parents who have experienced fetal or perinatal loss will have further pregnancies. Debate continues about recommendations concerning the optimum inter-pregnancy interval following such a death[6-9]. The WHO recommends couples wait at least six months before trying to conceive again[10], based on evidence that shorter inter-pregnancy intervals are associated with adverse pregnancy outcome[9, 11-13]. However, a recent meta-analysis found no clear evidence to support this recommendation[9], and UK guidance does not specify a waiting period. An additional concern is allowing sufficient time to grieve, and minimise the risk of trying to replace the deceased child, both of which have been associated with psychological and bonding issues[14,15, 16]. Hence, parents may receive contradictory advice from health professionals[17].

Many women experience an overwhelming urge to become pregnant as soon as possible after fetal or perinatal death[18-20]; 80% of women become pregnant within 18 months of the death[20-24]. The motives and processes involved in subsequent pregnancy decisions remain unclear. Health professional involvement in the decision to conceive is encouraged, and it is important for health professionals to listen to and support women where modifiable risks can be reduced to try and avert subsequent perinatal death[25, 26]. However, many women only inform health professionals once they have become pregnant[27-29], suggesting that the consideration of conception is primarily a personal decision between partners[19].

This systematic review aimed to identify, appraise and synthesise existing qualitative research reporting parents' experience of the decision-making process concerning becoming pregnant again after experiencing fetal or perinatal death.

Methods

Search strategy

Qualitative research reporting bereaved parents' inter-pregnancy experiences pertaining to thinking about, planning or preparing for subsequent pregnancy following a perinatal death

For peer review only - http://bmjopen.bmj.com/site/about/guidelines.xhtml

(miscarriage, stillbirth, termination of pregnancy for fetal anomaly (TOPFA) or neonatal death) was eligible for inclusion, if published in English in a peer-reviewed journal article. Search terms were identified using the SPIDER framework (Sample, Phenomenon of Interest, Design, Evaluation, Research Type)[30-32] (Appendix S1).

Electronic database searches (Medline, Web of Science, CINAHL, PsycINFO, ASSIA, Embase, PUBMED, Scopus and Google Scholar) were conducted between February and June 2018. Titles and abstracts were screened by ED using the web-based tool 'Rayyan'[33]. A 10% sample was blindly double screened, and any title highlighted as potentially relevant by either reviewer was included in the full-text review. There was very high agreement between screeners (97.2%) indicating a reduced risk of screening error. Two reviewers independently read all full text articles. Uncertainties about inclusion were resolved through discussion with a third reviewer. All included studies were also manually citation searched[34-39,40]. Fifteen studies were included in the final review (Appendix S2).

Quality appraisal

Study quality was assessed using the 2018 Critical Appraisal Skills Programme (CASP) checklist for qualitative research[41]. To help facilitate quality assessment, a scoring system was formulated so that numerical values were assigned to the three possible answers to the CASP questions (yes = 2, can't tell = 1, no = 0), with a maximum possible score of 20. Studies were considered 'good quality' if the overall score was 16 or more. Two reviewers independently rated each study, and differences were resolved by discussion. The quality score was considered during data analysis to ensure that all themes were present in better quality studies. Whilst there is a debate surrounding this approach, there is no "gold standard" tool for critical appraisal[42, 43]. As such, this pragmatic decision enabled the researchers to ascertain an indicative level of quality for all included papers which helped to mitigate the tension between reporting quality and relevance, an approach used successfully by other researchers[44].

Data extraction and synthesis

Data including bibliographic information, aims, method, quality assessment and findings were extracted onto an Excel form separately by two researchers to ensure consensus was reached. Qualitative data relating to first order (participants' quotes) and second order

For peer review only - http://bmjopen.bmj.com/site/about/guidelines.xhtml

constructs (author interpretations, assumptions, statements and ideas) were recorded and imported into QSR NVIVO 12 for management and thematic analysis.

Thematic analysis techniques, adapted from Butler *et al.,*[38] and Harden and Thomas[45], were used primarily by ED to systematically analyse and synthesise qualitative data from the included studies in two iterative stages:
Stage 1: Coding text: data from the primary studies were examined line by line for meaning and content, and within each study conceptual codes were systematically assigned allowing emerging concepts to be aggregated across the studies. Subsequent studies were coded into pre-existing concepts, and new concepts were created when deemed necessary.
Stage 2: Development of descriptive themes: the list of codes was examined and re-analysed for meaning and organised into categories. Each category was examined and descriptive themes were organised to reveal relationships and link theoretically similar sub-themes together, thus offering thought-provoking new insights into the body of knowledge in this area of research[46].

A priori themes of interest included: parents' decisions about pregnancy subsequent to loss, experiences surrounding preconception preparation and conception in the context of perinatal death, and the role of health professionals. All themes were independently checked for accuracy and reviewed by a second researcher to ensure a consensus was reached.

Patient and Public Involvement

Patients' and public views were not sought.

Results

Included studies

Fifteen studies published between 1986 and 2017 and conducted in eight countries met the inclusion criteria (Appendix S2, Table S1). Sample sizes ranged from four to 122 participants, and fetal or perinatal deaths occurred across a range of gestations. Investigating subsequent pregnancy decisions was the main aim of five studies[19, 21, 47-49], while ten studies reported relevant findings from studies where this was not the main focus. Seven studies focused solely on women's experiences[21, 47, 48, 50-53], seven included both parents[15, 16, 19, 49, 54-56] and one included wider family members. Participants predominantly reported ethnicity as

For peer review only - http://bmjopen.bmj.com/site/about/guidelines.xhtml

white[15, 16, 21, 52, 49, 56], relationship status as married or cohabiting[15, 16, 19, 21, 48-51, 53, 54] and had completed some form of post-secondary education[16, 48, 49, 54]. Twelve studies were considered 'good quality', including details of ethical approval, participant recruitment, research design, and evidence of researcher reflexivity (Table S2).

Findings

There were four main descriptive themes: 1) Deciding about subsequent pregnancy; 2) Diversity of personal reactions; 3) Social network influences; 4) Planning or timing of subsequent pregnancy. Ten subthemes were identified which interacted with the four themes (figure 1). Themes and subthemes were tabulated according to the studies from which they arose (Table S3).

Theme one: deciding about subsequent pregnancy

This theme captured findings relating to the immediate period after the loss, reflecting initial thoughts about subsequent pregnancies and the point at which pregnancy planning emerges as an idea. The subthemes highlight some of the complexity involved in decision making in the aftermath of perinatal death, especially when there is disagreement between partners.

Immediacy of thoughts

Eight studies reported data suggesting that parents form a clear idea about whether or not they wish to try to conceive again soon after the death of their baby[19, 21, 47, 49-51, 53, 55].

"Absolutely immediately…. Whilst we were waiting to be induced, we were talking about next time."[21]

The decision for a subsequent pregnancy may be influenced and made more difficult by a range of inter-related variables[16, 47, 49, 53]. These included whether there were existing children, the number of losses, age, experiences of pregnancy, relationship status and whether the pregnancy was planned. Initial decisions sometimes changed over time[49].

Differences in reactions between partners

Six studies considered the experiences of both parents; four found that parents sometimes disagreed on the decision to try for another baby[16, 19, 49, 54]. This was a key observation one study, where fathers were observed to be more reluctant to consider a future pregnancy[19].

For peer review only - http://bmjopen.bmj.com/site/about/guidelines.xhtml

"My husband was saying he didn't want any more children. So I don't know if you count this much but someone who has lost a baby, for me I would have been pregnant coming out of the hospital again I wanted to be pregnant again that badly and then he was saying he didn't want any more at all and he wouldn't discuss it until after we got the results from the hospital."[19]

In three studies[19, 53, 54], men were reported to carry a different burden to women, in particular taking on the role of 'protector' who needed to be emotionally strong[19, 53, 54], and less likely to express their own fears [19, 53, 54].

Whilst there is insufficient data here to support the idea that all parents know straight away about subsequent pregnancy, this theme is noteworthy because it highlights that parents may not simultaneously feel ready, and so access to specialised care to decide what is best for them may be required soon after the event, or may be required later on. This is in keeping with the findings from Conway and Russell[54], who emphasise the need to carry out more research into the most appropriate type and timing of intervention after loss.

Theme two: diversity of personal reactions

The subthemes represent how the unique and individual experiences and reactions of parents to loss is intrinsically linked in decisions surrounding the timing of subsequent pregnancy, which in turn influenced how each individual engaged with the concept of future pregnancy decisions.

Emotional response

Emotional responses to the death of a baby are unique to each parent, and varied from feelings of self-blame and guilt, loneliness and emptiness, anger, fear, failure and shame, to sadness and grief. In turn, the emotional response may not only influence, but also shape, understandings of the self and identity. In turn, these impact decisions about becoming pregnant after loss. In four studies, participants described the death as a void that could only be filled by a subsequent pregnancy[15, 21, 47, 55].

For peer review only - http://bmjopen.bmj.com/site/about/guidelines.xhtml

"Emotionally everything had been geared towards having a baby and then there was a big hole, a baby-shaped hole which was much bigger than a baby."[21]

Imagined family

Findings from eleven studies highlighted how pregnancy and childbirth are conceptualised within individuals' life narratives, and how parents often have pre-existing expectations about their reproductive aspirations. Experiencing a perinatal death prompts parents to reconsider their life goals[16, 19, 51].

"…but I was 29 when she was born, and I had this vision in my head that I was going to have all my kids by the time I was 30, and that wasn't working out, and so we better get on…."[16]

Easing perinatal grief

Five studies[21, 47, 49, 51, 55] reported that, for some parents, a subsequent pregnancy was considered essential for their recovery, eased feelings of perinatal grief, and gave hope for the future.

"I knew it was going to be a long process and it helped me recover because it made me focus on the future and I was more hopeful." [49]

However, not all parents were so certain that a subsequent pregnancy would aid recovery, and needed longer to overcome the grief and feel ready[19, 21, 47, 49]. These framings set the scene for future pregnancy planning, which emerged and developed with reference to how an individual has interpreted the meaning of the prior loss in relation to their sense of self.

This theme highlights just how individual and personal the decision to become pregnant is, and helps to make sense of why parents can react so differently to similar circumstances of loss. This necessarily colours the context in which questions about future pregnancy planning emerge, and develop, in ways that are highly dependent on how an individual has interpreted the meaning of the previous incidence of loss in relation to their sense of self.

For peer review only - http://bmjopen.bmj.com/site/about/guidelines.xhtml

Theme three: social network influences

Future pregnancy planning decisions could be influenced by an individual's social network and the cultural norms and expectations within their social group. Comments and assumptions by others about future planning could be interpreted as supportive or distressing, depending on the context.

Altered relationships and social networks

Eight studies[16, 19, 48, 50, 52, 55-57] highlighted how relationships and social networks changed after a perinatal death. In three studies, some parents reported much support thereby reinforcing bonds with their social network[21, 48, 56]. However, in five studies, parents felt that friends and family were not supportive, and relationships were profoundly altered following unhelpful attitudes and responses[19, 48, 50, 52, 56]. As a result, bereaved parents may withdraw or isolate themselves from friends, family and colleagues to avoid hurt[19, 48, 50, 52, 56], and avoid discussing their loss to prevent their friends and family feeling uncomfortable[56].

"Everything is okay with my friends, as long as I don't talk about my baby. When I do, they look away and change the subject. What am I supposed to do? I need to talk!"[56]

"[I] talked a lot about it with my boyfriend and friends. On the one hand it was fine, on the other hand not. None of my friends have experienced this, so it is quite difficult for them to understand. And then sometimes they said…well at least you know that you can become pregnant…I got that kind of remark."[48]

This subtheme highlights that the concept of subsequent pregnancy planning may be complicated by the comments and assumptions made by other within an individual's social network some of which could be interpreted as supportive, but in some circumstances, distressing.

Cultural norms and expectations

Nine studies discussed the impact of cultural norms on decisions about subsequent pregnancy[16, 19, 48, 50-53, 55, 56]. Parents in four studies noted other people's discomfort, reporting that such uneasiness led to unsupportive reactions[16, 21, 52, 56].

For peer review only - http://bmjopen.bmj.com/site/about/guidelines.xhtml

In some cultures, while there are strong expectations for women to bear children, stillbirth is regarded as taboo and parents are not expected to see or to discuss their dead baby[50, 51]. A striking finding was the guilt and sense of failure that was reported following a perinatal death in this environment[50, 51]. This led some women to pursue pregnancy as soon as possible to undo the sense of wrongdoing associated with stillbirth[50] and fulfil the cultural expectations of being a successful mother[51].

"I am trying to have a baby again. As long as I can have a baby, my mother-in-law can't look down on me. Also, failing to deliver a baby makes me feel that I am not a woman. Every woman should be able to deliver a baby – that's what makes a woman so different from a man."[50]

This theme highlights that the concept of subsequent pregnancy planning may be complicated by the comments and assumptions made by others within an individual's social network. Pregnancy and childbirth may be viewed by society as a natural and celebratory part of life, rather than the anxiety-filled prospect that bereaved parents now face. Some comments could be interpreted as supportive, but some parents may perceive the expectation for them to 'get over it' and 'get on with it' hard to tolerate, especially as some felt that a subsequent pregnancy could be dismissive of their deceased baby.

Theme four: planning or timing subsequent pregnancy

Encapsulated in this theme is the notion that planning or timing of a subsequent pregnancy may be influenced by the advice, information and reassurance from health professionals, which may differ depending on the emotional and medical barriers faced by bereaved parents after loss.

Eleven studies reported discussion of planning or timing a subsequent pregnancy[16, 19, 21, 47-49, 52, 54-57]. In six studies, parents felt that the timing of a subsequent pregnancy should be based on personal reasons and individual experience, rather than the thoughts of others, including medical professionals[19, 21, 47, 49, 52, 56]. Women spoke of listening to their body and trusting their own feelings[21, 47]. Planning a subsequent pregnancy often involved overcoming barriers[16, 47, 49, 55, 57], and sometimes conflicting messages from health professionals[56].

For peer review only - http://bmjopen.bmj.com/site/about/guidelines.xhtml

Barriers – practical and emotional

In five studies, parents cited a range of emotional and medical challenges as barriers to conceiving after loss[16, 47, 49, 55, 57]. Parents faced a period of ambiguity and uncertainty when planning their next pregnancy[16, 19, 21, 48, 49, 55, 57], and a loss of control associated with a long conception period[48].

"The last time I was actually very impatient because after that last miscarriage it lasted a year and a half before we were pregnant again. So I thought it would take a year and a half again to become pregnant so we tried again a month after the miscarriage." [48]

Such uncertainty was intensified by practical factors, such as the number of previous perinatal deaths experienced, advanced maternal age, financial strain, parent relationship status and fertility problems[48, 49, 57]. High levels of anxiety and fear were linked to the lack of reassurance that a subsequent pregnancy would end successfully[16, 19, 52, 54, 55, 57].

Such barriers may redefine decisions about how many children parents wish to have[57], or how soon parents try to conceive, with some deciding to delay to make sure they felt emotionally and physically ready for pregnancy[21, 47-49, 55], whereas others may try to become pregnant regardless of emotional or physical readiness for fear of not being able to conceive again[16].

Mother: "I also had a real fear of not becoming pregnant again, I thought that maybe that was my once in a lifetime shot. So that was on my mind too, that maybe I wouldn't get pregnant. So after 6 months I was real anxious to get going because I thought it might take a while.

Father: Well, we missed one month, and of course right away L. panicked and figured out she'd never become pregnant again."[16]

Health professional advice

Seven studies discussed the involvement of health professionals in the inter-pregnancy period. The amount and type of advice given varied, but included answering medical questions, providing information and advice about subsequent pregnancy and reassurance about the level of care that would be received for a subsequent pregnancy[16, 19, 47-49, 54, 56].

For peer review only - http://bmjopen.bmj.com/site/about/guidelines.xhtml

One early study focused specifically on mothers' perceptions of medical advice about timing of a subsequent pregnancy[47]. Again, the advice varied; five women were advised to wait less than six months before trying to conceive, matching the women's expectations, as they felt an urgent need to get pregnant[47]. Fourteen were advised to wait at least six months, 11 of whom found this advice unacceptable mainly due to the strong desire to have a baby:

"[12 months] is an eternity....I had all this parenting energy and nowhere to direct it…." [47]

Five women in this study received no *specific* advice about waiting. These women appreciated this both at the time and in hindsight, as it empowered them to make their own informed decision based on their individual needs. This was a consistent finding in other studies[21, 47, 49, 56] and highlights the importance of *appropriate* advice, rather than the amount or the content of the advice.

Health professional information and reassurance

Concerned parents sought reassurance and information from health professionals, for example, about the risks of another negative outcome and the type of specialised care and emotional support they might expect to receive in a future pregnancy[16, 19, 21, 47, 49, 52, 55, 56].

"…they basically told us that we had the same chance of it happening to us again as if it had never happened to us. The same as any couple walking down the street. But the only thing I kept saying was that it did happen to us you know so they can't give any reassurances."[19]

However, not all parents received such advice and support[21], and although parents can be informed of level of risk, it was not possible to guarantee that there will not be another negative outcome in a future pregnancy[19].

This theme highlights how bereaved parents may turn to health professionals for advice, information and reassurance regarding the timing of a subsequent pregnancy and overcoming barriers to conception.

For peer review only - http://bmjopen.bmj.com/site/about/guidelines.xhtml

Discussion

This review analysed and synthesised qualitative data from primary studies, describing the experiences of bereaved parents in their transition from perinatal death to pregnancy, an area largely overlooked in the literature to date. The sample included a range of experiences from both parents, across eight countries, and included parents who had experienced different types of fetal or perinatal death. The themes identified were supported by evidence grounded in the primary studies. This systematic review followed the Enhancing Transparency in Reporting the Synthesis of Qualitative Research (ENTREQ) reporting structure (Appendix S3). To reduce the risk of bias, we used standardised and comprehensive methods, with explicit criteria, double blind screening, quality rating and data extraction. As researchers, not practitioners in the field, we have a particular interest in understanding both lay and professional experiences of health care provided in the context of distressing life events such as reproductive loss. It was therefore important to stay close to the data so as not to impart our own opinions or judgements on the findings.

Many parents decide whether or not they wanted to have another pregnancy shortly after the loss. However, parents sometimes disagreed with their partners and early decisions could evolve over time. A key theme was the range of unique and personal reactions to loss. These experiences were intrinsically linked to decisions surrounding the timing of subsequent pregnancy, which in turn influenced how participants engaged with future pregnancy decision-making. Future pregnancy planning may be influenced by an individual's social network and the cultural norms that guide people within their social group. The taboo that surrounds perinatal death makes it a difficult subject to discuss, potentially leaving bereaved parents feeling isolated and lonely. A salient finding was the assertion that parents should be provided with information about timing subsequent pregnancy, rather than prescriptive advice or specific recommendations[21, 47, 49, 56]. Health professionals need to be mindful of the patients' individual preference for the amount and type of advice that they want or need. Parents should be empowered to access information at a time of their choosing, when they feel ready.

Resource constraints limited the review to journal articles published in English. Relevant material was identified in other sources including abstracts and theses, and some themes may not have been identified owing to the large number of search results and the fact that relevant studies may report data as secondary findings, increasing the likelihood of screening error

For peer review only - http://bmjopen.bmj.com/site/about/guidelines.xhtml

resulting in studies being missed. The majority of study participants were mothers; fathers' experiences may be underrepresented. All studies were undertaken in high income countries, and participants were mainly educated and living with the father of the baby. This has implications for the wider applicability of the findings.

There are inherent limitations associated with systematically reviewing qualitative studies, since only data which has been selected for presentation within the study is available, rather than the totality of the data collected. The quality of a systematic review further depends on the quality of the studies it includes. While the majority of the included studies were rated as good quality, not all studies indicated the data analysis technique used or the retrospectivity of loss, which may impact the reliability of findings. Care was taken to ensure that themes were represented across multiple studies, one study rated as average quality contributed to six of the ten subthemes and dominated the findings in the subtheme regarding health professional advice. Thus, caution should be exercised regarding the wider applicability of this theme.

The findings suggested that some parents develop a clear idea soon after perinatal death whether or not they wish to pursue a subsequent pregnancy. This reaction to pregnancy loss may reflect a strong desire to leave the liminal phase that parents experience following the death of a baby, whereby the nebulous identity of becoming non-pregnant leaves parents stranded between the stable states of being pregnant and parenthood[56, 58]. Other explanations include the theory that mothers and fathers assume their role of parents early in pregnancy[59], with psychological preparation for parenthood beginning before conception[60]. Jaffe and Diamond further suggest that people determine their own 'reproductive stories' as early as their own childhood; the perinatal death leaves this desire unfulfilled until a living child is born[61]. Whilst there is insufficient data to conclude that all parents decided quickly about subsequent pregnancy, we suggest that some parents may welcome opportunities to discuss their plans earlier than health professionals may assume. Conway and Russell emphasise the need to understand the most appropriate type and timing of health professional intervention after loss[54].

The data described a range of reactions to loss, and highlight the personal and individual nature of timing a subsequent pregnancy. This is consistent with other studies[18, 62-67 68 64]. Our findings suggest that parents may feel increased isolation after fetal or perinatal death,

For peer review only - http://bmjopen.bmj.com/site/about/guidelines.xhtml

due to the taboo nature of the topic[18, 69, 70]. Social withdrawal has also been identified as an expression of grief following bereavement[71, 72].

The needs of bereaved parents during the period when they are contemplating becoming pregnant again has received substantially less interest than the need for additional support during subsequent pregnancy. Several studies have explored the experiences of parents during pregnancy following loss, and services have been developed to respond to their need for increased support. The process of conceiving the subsequent pregnancy is not discussed in these reports[22, 28, 73-75]. It is possible that health professionals do not consider involvement in such decisions as part of their role. However, there are a number of causes or risk factors for fetal and perinatal death which may be amenable to intervention in the inter-pregnancy interval, for example diabetes, obesity or smoking.

This review highlights that the window of opportunity to influence these factors and support parents in reducing the risks in the next pregnancy may be relatively short. Health professionals may play an important role as a bridge between medical information and emotional support in the inter-pregnancy interval, but with a paucity of research looking specifically at the perspectives of health professionals it is not possible to establish why advice may differ between health professionals and whether they feel able to offer the individualised advice and support that bereaved parents require or seek.

Conclusion

Many parents think about becoming pregnant again very soon after experiencing a pregnancy loss, and health professionals should anticipate the need to facilitate conversations from the very earliest point. Providing personalised and flexible support may be challenging for health professionals working within healthcare systems which may not easily adapt to differing needs. Health professional perspectives are currently under-analytically developed in the literature, so should be an area for further development. Further research should also address the need to better understand the decision-making process, support requirements and challenges that parents face in the inter-pregnancy interval following pregnancy loss, and address how bereavement services can integrate better with pregnancy preparation services. This could be of particular benefit to those with medical conditions, such as pre-existing diabetes, where effective preparation for pregnancy and delayed conception have been shown to significantly improve outcomes[76, 77].

For peer review only - http://bmjopen.bmj.com/site/about/guidelines.xhtml

4562

Disclosure of interests All authors confirm that they have no interests to declare.

Contribution to authorship ED contributed to protocol development, performed the searches, reviewed the papers, conducted the thematic synthesis and wrote the first draft of the article. RB developed the idea for the study, contributed to protocol development, reviewed papers for inclusion, quality assessment and data extraction, and contributed to writing the final article. RG developed the idea for the study, contributed to protocol development, reviewed papers for inclusion, quality assessment and data extraction, supervised and reviewed the thematic synthesis and contributed to writing the final article. JR developed the idea for the study, contributed to protocol development, reviewed papers for inclusion, quality assessment and data extraction, and contributed to writing the final article

Details of ethics approval No ethical approval required.

PROSPERO registration: CRD42018112839

Funding: This work was supported by the Economic and Social Research Council [grant number ES/P000762/1].

Data availability: All data used in the analysis is available in the published papers. Additional data available from the authors comprises the text extracted from the papers relevant to the review question and used in the thematic analysis.

For peer review only

References

1 World Health Organization. Neonatal and perinatal mortality : country, regional and global estimates. Geneva: World Health Organization 2006.

2 Fretts RC. Etiology and prevention of stillbirth. American Journal of Obstetrics and Gynecology. 2005;193(6):1923-35.

3 Office for National Statistics. Childhood, Infant and Perinatal Mortality in England and Wales 2015. 2015 [cited 20 November 2017]; Available from: https://www.ons.gov.uk/peoplepopulationandcommunity/birthsdeathsandmarriages/deaths/bulletins/childhoodinfantandperinatalmortalityinenglandandwales/2015

4 Statham H, Green JM. The effects of miscarriage and other "unsuccessful" pregnancies on feelings early in a subsequent pregnancy. Journal of Reproductive and Infant Psychology. 1994 Jan-Mar;12(1):45-54.

5 Woods JR, Woods JLE. Loss during pregnancy or in the newborn period. Pitman, New Jersey: Jannetti Publications 1997.

6 Love ER, Bhattacharya S, Smith NC, Bhattacharya S. Effect of interpregnancy interval on outcomes of pregnancy after miscarriage: retrospective analysis of hospital episode statistics in Scotland. BMJ. 2010;341.

7 Fockler ME, Ladhani NNN, Watson J, Barrett JFR. Pregnancy subsequent to stillbirth: Medical and psychosocial aspects of care. Seminars in Fetal and Neonatal Medicine. 2017;22(3):186-92.

8 Gold KJ, Leon I, Chames MC. National survey of obstetrician attitudes about timing the subsequent pregnancy after perinatal death. American Journal of Obstetrics and Gynecology. 2010;202(4):357.

9 Kangatharan C, Labram S, Bhattacharya S. Interpregnancy interval following miscarriage and adverse pregnancy outcomes: systematic review and meta-analysis. Human Reproduction Update. 2017;23(2):221-31.

10 World Health Organization. Report of a WHO Technical Consultation on Birth Spacing. Geneva 2005.

11 Conde-Agudelo A, Rosas-Bermudez A, Castaño F, Norton MH. Effects of Birth Spacing on Maternal, Perinatal, Infant, and Child Health: A Systematic Review of Causal Mechanisms. Studies in Family Planning. 2012;43(2):93-114.

12 Buchmayer SM, Sparén P, Cnattingius S. Previous pregnancy loss: Risks related to severity of preterm delivery. American Journal of Obstetrics and Gynecology. 2004;191(4):1225-31.

13 Bhattacharya S, Townend J, Shetty A, Campbell D, Bhattacharya S. Does miscarriage in an initial pregnancy lead to adverse obstetric and perinatal outcomes in the next continuing pregnancy? BJOG: An International Journal of Obstetrics & Gynaecology. 2008;115(13):1623-9.

14 Hughes PM, Turton P, Evans CD. Stillbirth as risk factor for depression and anxiety in the subsequent pregnancy: cohort study. BMJ. 1999;318(7200):1721-4.

15 Grout LA, Romanoff BD. The myth of the replacement child: parents' stories and practices after perinatal death. Death Studies. 2000;24(2):93-113.

16 Phipps S. The subsequent pregnancy after stillbirth: Anticipatory parenthood in the face of uncertainty. The International Journal of Psychiatry in Medicine. 1986;15(3):243-64.

17 Goldstein RR, Croughan MS, Robertson PA. Neonatal outcomes in immediate versus delayed conceptions after spontaneous abortion: a retrospective case series. American Journal of Obstetrics and Gynecology. 2002;186(6):1230-4; discussion 4-6.

18 Burden C, Bradley S, Storey C, Ellis A, Heazell AE, Downe S, et al. From grief, guilt pain and stigma to hope and pride - a systematic review and meta-analysis of mixed-method research of the psychosocial impact of stillbirth. BMC Pregnancy & Childbirth. 2016;16(9):1-12.

For peer review only - http://bmjopen.bmj.com/site/about/guidelines.xhtml

19 Meaney S, Everard CM, Gallagher S, O'Donoghue K. Parents’ concerns about future pregnancy after stillbirth: a qualitative study. Health Expectations. 2017;20(4):555-62.

20 Forrest GC, Standish E, Baum JD. Support after perinatal death: a study of support and counselling after perinatal bereavement. BMJ. 1982;285(6353):1475-9.

21 Lee L, McKenzie-McHarg K, Horsch A. Women's Decision Making and Experience of Subsequent Pregnancy Following Stillbirth. Journal of Midwifery & Women's Health. 2013;58(4):431-9.

22 DeBackere KJ, Hill PD, Kavanaugh KL. The Parental Experience of Pregnancy After Perinatal Loss. Journal of Obstetric, Gynecologic, & Neonatal Nursing. 2008;37(5):525-37.

23 Mills TA, Ricklesford C, Cooke A, Heazell AE, Whitworth M, Lavender T. Parents' experiences and expectations of care in pregnancy after stillbirth or neonatal death: a metasynthesis. BJOG: An International Journal of Obstetrics & Gynaecology. 2014;121(8):943-50.

24 Cuisinier M, Janssen H, De Graauw C, Bakker S, Hoogduin C. Pregnancy following miscarriage: course of grief and some determining factors. Journal of Psychosomatic Obstetrics & Gynecology. 1996;17(3):168-74.

25 August EM, Salihu HM, Weldeselasse H, Biroscak BJ, Mbah AK, Alio AP. Infant mortality and subsequent risk of stillbirth: a retrospective cohort study. Bjog. 2011 Dec;118(13):1636-45.

26 Monari F, Facchinetti F. Management of subsequent pregnancy after antepartum stillbirth. A review. The Journal of Maternal-Fetal & Neonatal Medicine. 2010;23(10):1073-84.

27 Robson SJ, Leader LR. Management of subsequent pregnancy after an unexplained stillbirth. Journal of Perinatology. 2010;30:305-10.

28 Lamb EH. The Impact of Previous Perinatal Loss on Subsequent Pregnancy and Parenting. The Journal of Perinatal Education. 2002;11(2):33-40.

29 Robson SJ, Leader LR, Dear KBG, Bennett MJ. Women’s expectations of management in their next pregnancy after an unexplained stillbirth: An Internet-based empirical study. Australian and New Zealand Journal of Obstetrics and Gynaecology. 2009;49(6):642-6.

30 Cooke A, Smith D, Booth A. Beyond PICO:The SPIDER Tool for Qualitative Evidence Synthesis. Qualitative Health Research. 2012;22(10):1435-43.

31 Stern C, Jordan, Z., McArthur, A. Developing the Review Question and Inclusion Criteria. AJN: The American Journal of Nursing. 2014;114(4):53-6.

32 Methley AM, Campbell S, Chew-Graham C, McNally R, Cheraghi-Sohi S. PICO, PICOS and SPIDER: a comparison study of specificity and sensitivity in three search tools for qualitative systematic reviews. BMC Health Services Research. 2014;14(1):579-89.

33 Ouzzani M, Hammady H, Fedorowicz Z, Elmagarmid A. Rayyan — a web and mobile app for systematic reviews. Systematic Reviews. 2016;5(210).

34 Dixon-Woods M, Bonas S, Booth A, Jones DR, Miller T, Sutton AJ, et al. How can systematic reviews incorporate qualitative research? A critical perspective. Qualitative Research 2006;6(1):27-44.

35 Evans D. Database searches for qualitative research. Journal of the Medical Library Association. 2002;90(3):290-3.

36 Jones ML. Application of systematic review methods to qualitative research: practical issues. Journal of Advanced Nursing. 2004;48(3):271-8.

37 Dixon-Woods M, Sutton A, Shaw R, Miller T, Smith J, Young B, et al. Appraising qualitative research for inclusion in systematic reviews: a quantitative and qualitative comparison of three methods. Journal of Health Services Research & Policy. 2007;12(1):42-7.

38 Butler A, Hall H, Copnell B. A guide to writing a qualitative systematic review protocol to enhance evidence-based practice in nursing and health care. Worldviews on Evidence-Based Nursing. 2016;13(3):241-9.

39 McKibbon KA, Wilczynski NL, Haynes RB. Developing Optimal Search Strategies for Retrieving Qualitative Studies in PsycINFO. Evaluation & the Health Professions. 2006;29(4):440-54.

For peer review only - http://bmjopen.bmj.com/site/about/guidelines.xhtml

40 Lockwood C, Porrit K, Munn Z, Rittenmeyer L, Salmond S, Bjerrum M, Loveday H, Carrier J, Stannard D. Systematic reviews of qualitative evidence. In: Aromataris EM, Z. , ed. *Joanna Briggs Institute Reviewer's Manual*: The Joanna Briggs Institute 2017.
41 Critical Appraisal Skills Programme (CASP). Qualitative Checklist 2018 [cited 10th September 2018]; Available from: https://casp-uk.net/wp-content/uploads/2018/01/CASP-Qualitative-Checklist.pdf
42 Katrak P, Bialocerkowski AE, Massy-Westropp N, Kumar VSS, Grimmer KA. A systematic review of the content of critical appraisal tools. BMC Medical Research Methodology. 2004 2004/09/16;4(1):22.
43 Soilemezi D, Linceviciute S. Synthesizing Qualitative Research:Reflections and Lessons Learnt by Two New Reviewers. International Journal of Qualitative Methods. 2018;17(1):1609406918768014.
44 Feder GS, Hutson M, Ramsay J, Taket AR. Women exposed to intimate partner violence: expectations and experiences when they encounter health care professionals: a meta-analysis of qualitative studies. Archives of internal medicine. 2006 Jan 9;166(1):22-37.
45 Harden A, Thomas J. Methodological issues in combining diverse study types in systematic reviews. International Journal of Social Research Methodology. 2005;8(3):257-71.
46 Bearman M. Quality and literature reviews: beyond reporting standards. Medical Education. 2016;50(4):382-4.
47 Davis DL, Stewart M, Harmon RJ. Postponing Pregnancy after Perinatal Death: Perspectives on Doctor Advice. Journal of the American Academy of Child & Adolescent Psychiatry. 1989;28(4):481-7.
48 Ockhuijsen HDL, van den Hoogen A, Boivin J, Macklon NS, de Boer F. Pregnancy After Miscarriage: Balancing Between Loss of Control and Searching for Control. Research in Nursing & Health. 2014;37(4):267-75.
49 Keim MC, Fortney CA, Shultz EL, Winning A, Gerhardt CA, Baughcum A. Parent Distress and the Decision to Have Another Child After an Infant's Death in the NICU. JOGNN: Journal of Obstetric, Gynecologic & Neonatal Nursing. 2017;46(3):446-55.
50 Hsu M, Tseng Y, Kuo L. Transforming loss: Taiwanese women's adaptation to stillbirth. Journal of Advanced Nursing. 2002;40(4):387-95.
51 Tseng YF, Chen CH, Wang HH. Taiwanese women's process of recovery from stillbirth: a qualitative descriptive study. Research in Nursing & Health. 2014;37(3):219-28.
52 Bansen SS, Stevens HA. Women's experiences of miscarriage in early pregnancy. Journal of Nurse-Midwifery. 1992;37(2):84-90.
53 Cecil R. "I wouldn't have minded a wee one running about": Miscarriage and the family. Social Science & Medicine. 1994;38(10):1415-22.
54 Conway K, Russell G. Couples' grief and experience of support in the aftermath of miscarriage. British Journal of Medical Psychology. 2000;73(4):531-45.
55 Carlsson T, Bergman G, Karlsson A-M, Wadensten B, Mattsson E. Experiences of termination of pregnancy for a fetal anomaly: A qualitative study of virtual community messages. Midwifery. 2016;41:54-60.
56 de Montigny F, Dumas L, Beaudet L. A baby has died: The impact of perinatal loss on family social networks. Journal of Obstetric, Gynecologic & Neonatal Nursing. 1999;28(2):151-6.
57 Cacciatore J, DeFrain J, Jones KLC. When a Baby Dies: Ambiguity and Stillbirth. Marriage & Family Review. 2008;44(4):439-54.
58 Reiheld A. "The Event That Was Nothing": Miscarriage as a Liminal Event. Journal of Social Philosophy. 2015;46(1):9-26.
59 Rubin R. Attainment of the maternal role: Part 1. Processes. Nursing Research. 1967;16(3):237-45.
60 Shapiro CH. Infertility and pregnancy loss: A guide for helping professionals. 1988;251.

For peer review only - http://bmjopen.bmj.com/site/about/guidelines.xhtml

61 Jaffe J, Diamond MO. When the reproductive story goes awry: Trauma and loss. *Reproductive Trauma : Psychotherapy with Infertility and Pregnancy Loss Clients*. Washington DC, US: The American Psychology Association 2011.
62 Brooten D, Youngblut JM, Hannan J, Caicedo C, Roche R, Malkawi F. Infant and child deaths: Parent concerns about subsequent pregnancies. Journal of the American Association of Nurse Practitioners. 2015;27(12):690-7.
63 Koopmans L, Wilson T, Cacciatore J, Flenady V. Support for mothers, fathers and families after perinatal death. Cochrane Database of Systematic Reviews. 2013(6).
64 Ellis A, Chebsey C, Storey C, Bradley S, Jackson S, Flenady V, et al. Systematic review to understand and improve care after stillbirth: a review of parents' and healthcare professionals' experiences. BMC Pregnancy and Childbirth. 2016 January 25;16(1):16.
65 Erlandsson K, Lindgren H, Malm MC, Davidsson-Bremborg A, Rådestad I. Mothers' experiences of the time after the diagnosis of an intrauterine death until the induction of the delivery: a qualitative Internet-based study. The journal of obstetrics and gynaecology research. 2011;37(11):1677-84.
66 Trulsson O, Rådestad I. The silent child - mothers' experiences before, during, and after stillbirth. Birth: Issues in Perinatal Care. 2004;31(3):189-95.
67 Malacrida CA. Perinatal death: helping parents find their way. Journal of Family Nursing. 1997;3(2):130-48.
68 Turton P, Badenhorst W, Hughes P, Ward J, Riches S, White S. Psychological impact of stillbirth on fathers in the subsequent pregnancy and puerperium. The British Journal of Psychiatry. 2006 Feb;188:165-72.
69 Hutti MH. An exploratory study of the miscarriage experience. Health Care for Women International. 1986;7(5):371-89.
70 Swanson-Kauffman KM. Caring in the instance of unexpected early pregnancy loss. Topics in clinical nursing. 1986;8(2):37-46.
71 Asplin N, Wessel H, Marions L, Öhman SG. Pregnancy termination due to fetal anomaly: Women's reactions, satisfaction and experiences of care. Midwifery. 2014;30(6):620-7.
72 Capitulo KL. Evidence for healing interventions with perinatal bereavement. MCN: The American Journal of Maternal/Child Nursing. 2005;30(6):389-96.
73 Côté-Arsenault D, Marshall R. One Foot In-One Foot Out: Weathering the Storm of Pregnancy After Perinatal Loss. Research in Nursing & Health. 2000;23(6):473-85.
74 Hunter A, Tussis, L., MacBeth, A. The presence of anxiety, depression and stress in women and their partners during pregnancies following perinatal loss: A meta-analysis. Journal of Affective Disorders. 2017;223(Supplement C):153-64.
75 Mills TA, Ricklesford C, Heazell AEP, Cooke A, Lavender T. Marvellous to mediocre: findings of national survey of UK practice and provision of care in pregnancies after stillbirth or neonatal death. BMC Pregnancy and Childbirth. 2016;16(101).
76 McElvy SS, Miodovnik M, Rosenn B, Khoury JC, Siddiqi T, Dignan PSJ, et al. A focused preconceptional and early pregnancy program in women with type 1 diabetes reduces perinatal mortality and malformation rates to general population levels. Journal of Maternal-Fetal Medicine. 2000;9(1):14-20.
77 Murphy HR, Roland JM, Skinner TC, Simmons D, Gurnell E, Morrish NJ, et al. Effectiveness of a regional prepregnancy care program in women with type 1 and type 2 diabetes: benefits beyond glycemic control. Diabetes Care. 2010;33(12):2514-20.

For peer review only - http://bmjopen.bmj.com/site/about/guidelines.xhtml

Figure 1: Themes and subthemes

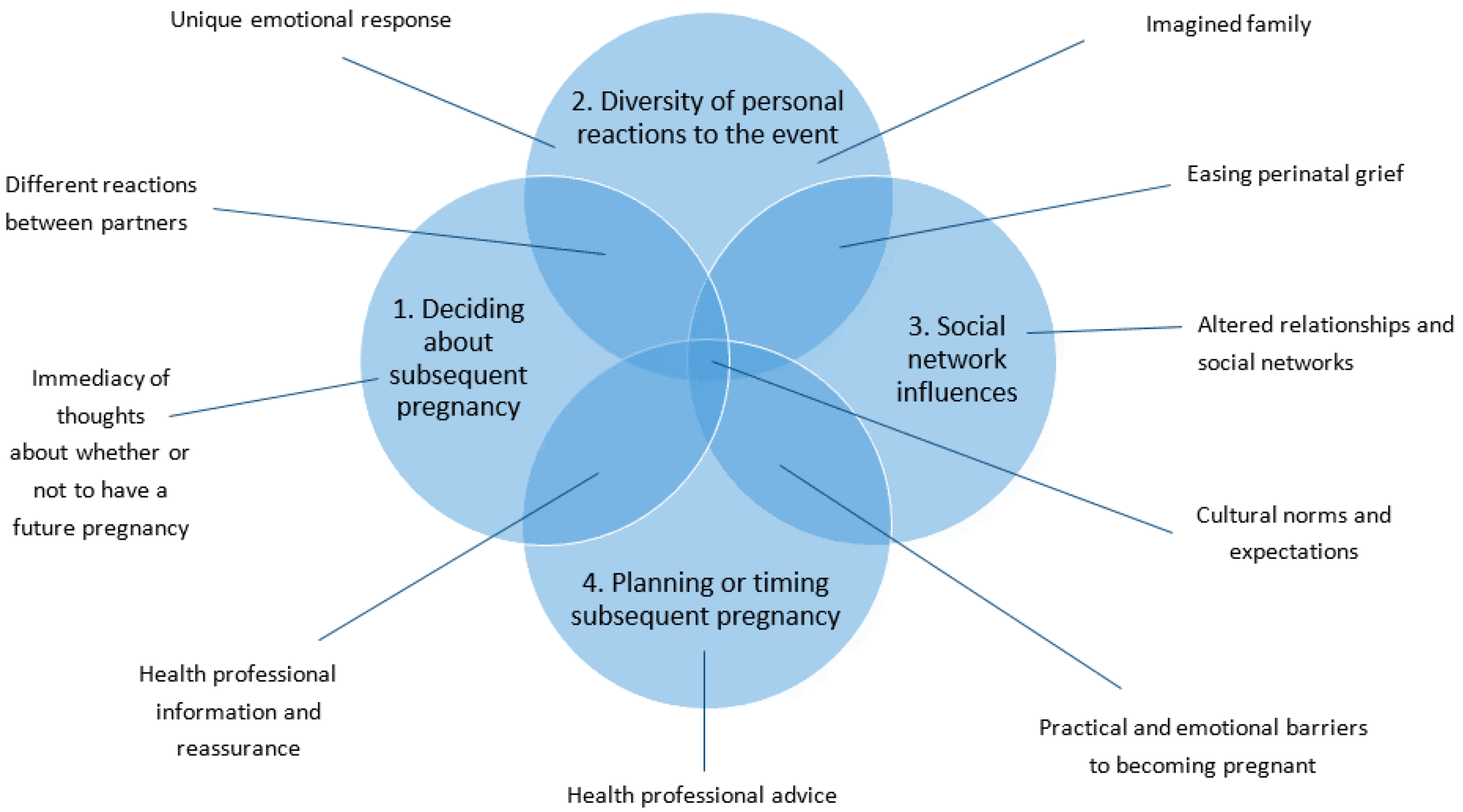

For peer review only

Appendix S1

	Search Terms
1.	(Stillbirth or still-birth or "perinatal loss" or "perinatal death" or "perinatal mortality" or "neonatal death" or miscarriage or "reproductive loss" or "pregnancy complications" or "fetal death" or "fetal resorption" or "foetal death" or "foetal resorption").mp.
2.	(Infant Mortality or Pregnancy outcome or Fetal Diseases or Foetal Diseases or Pregnancy Complications).mp.
3.	(baby death or newborn death or new-born death).mp.
4.	(abortion, spontaneous or abortion or “termination of pregnancy”).mp. [mp=title, abstract, original title, name of substance word, subject heading word, keyword heading word, protocol supplementary concept word, rare disease supplementary concept word, unique identifier, synonyms]
5.	(behavior or behaviour or emotion* or psychosocial or "emotional state" or "psychological state").mp. [mp=title, abstract, original title, name of substance word, subject heading word, keyword heading word, protocol supplementary concept word, rare disease supplementary concept word, unique identifier, synonyms]
6.	(bereavement or grief or expressed emotion).mp.
7.	(experience* or perception* or feeling* or attitude* or voice*).mp.
8.	(pregnancy or pre-pregnancy or "subsequent pregnancy" or "previous pregnancy" or "past pregnancy").mp.
9.	(pre-conception or preconception or "preconception care" or "prenatal care").mp.
10.	(peri-conception or periconception).mp.
11.	(interpregnancy or inter-pregnancy or "interpregnancy interval" or "inter-pregnancy interval").mp.
12.	("Birth Interval*" or "birth spacing").mp. [mp=title, abstract, original title, name of substance word, subject heading word, keyword heading word, protocol supplementary concept word, rare disease supplementary concept word, unique identifier, synonyms]
13.	(contraception or "contraception behavio*" or interconception or inter-conception or postpartum or post-partum).mp.
14.	(((Decision/ adj4 pregnanc*.mp.) or plan*.mp.) adj3 pregnancy.mp.) or "family planning".mp. [mp=title, abstract, original title, name of substance word, subject heading word, keyword heading word, protocol supplementary concept word, rare disease supplementary concept word, unique identifier, synonyms]
15.	(parent* or father or mother* or wom*n or "single parent" or spouse* or relationship* or "partner relationship*").mp. [mp=title, abstract, original title, name of substance word, subject heading word, keyword heading word, protocol supplementary concept word, rare disease supplementary concept word, unique identifier, synonyms]
16.	(psychosocial or psychological state).mp.
17.	(mixed-method or multi-method).mp. or empirical research/ or grounded theory/ or qualitative research/ or hermeneutics/ or interdisciplinary research/ or phenomenological research.mp. or phenomenology.mp. [mp=title, abstract, original title, name of substance word, subject heading word, keyword heading word, protocol supplementary concept word, rare disease supplementary concept word, unique identifier, synonyms]
18.	(interview or focus group or survey or questionnaire or diar* or cohort stud* or case stud* or ethnograph* or retrospective or longitudinal or observation*).mp. [mp=title, abstract, original title, name of substance word, subject heading word, keyword heading word, protocol supplementary concept word, rare disease supplementary concept word, unique identifier, synonyms]
19.	17 or 18
20.	1 or 2 or 3 or 4
21.	5 or 6 or 7 or 14 or 16
22.	8 or 9 or 10 or 11 or 12 or 13
23.	15 and 19 and 20 and 21 and 22
24.	limit 23 to (abstracts and english language and humans and "qualitative (maximizes sensitivity)")
25.	20
26.	limit 20 to abstracts
27.	15 and 19 and 21 and 22 and 26
28.	limit 27 to (english language and humans and "qualitative (maximizes specificity)")

For peer review only

Appendix S2

PRISMA flow chart of search strategy and outcomes.

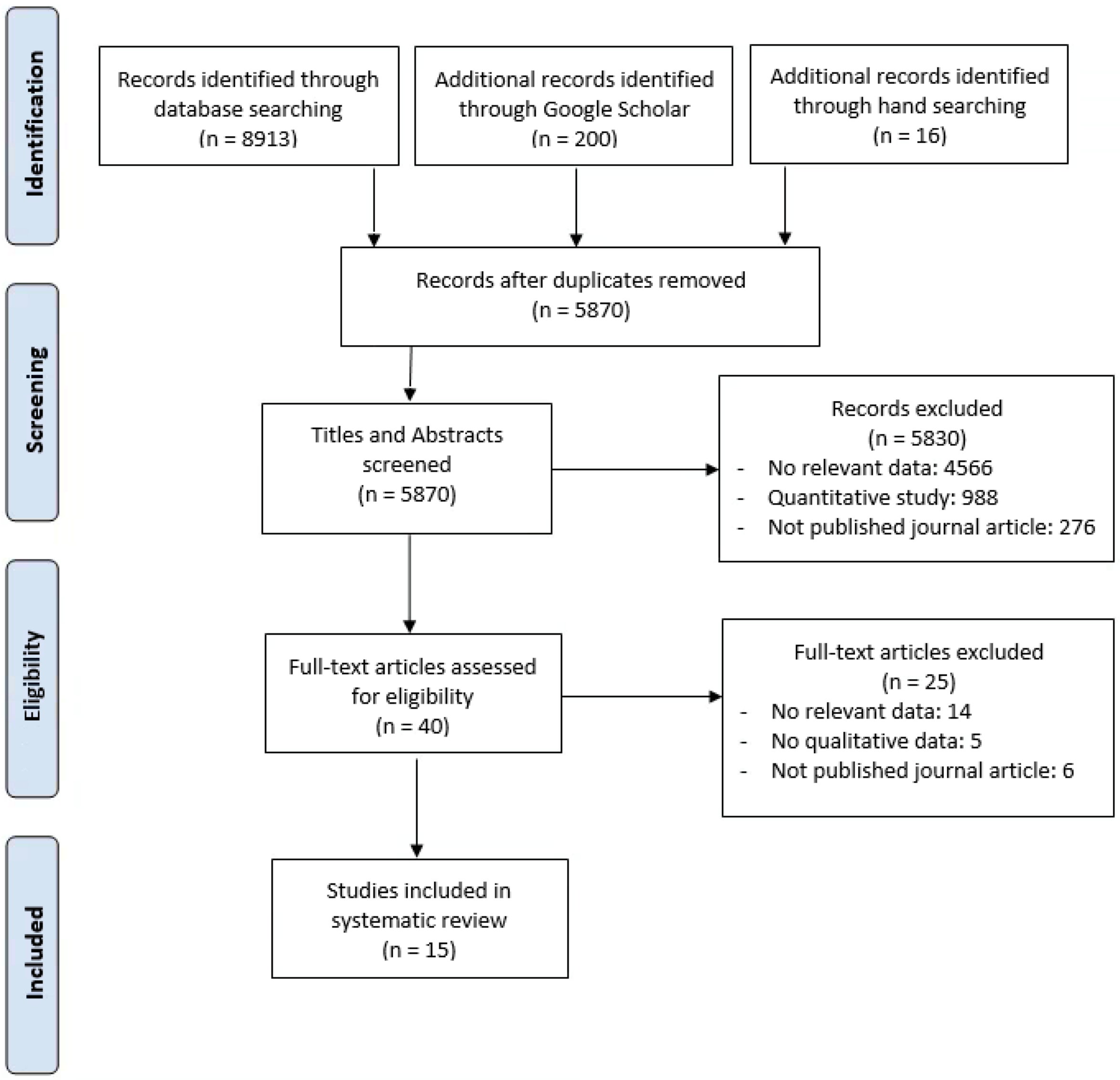

ENTREQ Reporting Template

Number	Item	Description	Page/Line Number
1	**Aim**	State the research question the synthesis addresses.	Page 3, line 87-89
2	**Synthesis methodology**	Identify the synthesis methodology or theoretical framework which underpins the synthesis, and describe the rationale for choice of methodology *(e.g. meta-ethnography, thematic synthesis, critical interpretive synthesis, grounded theory synthesis, realist synthesis, meta-aggregation, meta-study, framework synthesis).*	Page 5, lines 131-147
3	**Approach to searching**	Indicate whether the search was pre-planned (comprehensive search strategies to seek all available studies) or iterative (to seek all available concepts until they theoretical saturation is achieved).	Page 3, lines 93-94<br>Page 4, lines 95-108
4	**Inclusion criteria**	Specify the inclusion/exclusion criteria *(e.g. in terms of population, language, year limits, type of publication, study type).*	Page 3, lines 93-94<br>Appendix S1
5	**Data sources**	Describe the information sources used (e.g. *electronic databases (MEDLINE, EMBASE, CINAHL, psycINFO, Econlit), grey literature databases (digital thesis, policy reports), relevant organisational websites, experts, information specialists, generic web searches (Google Scholar) hand searching, reference lists)* and when the searches conducted; provide the rationale for using the data sources.	Page 4, lines 100-102
6	**Electronic search strategy**	Describe the literature search *(e.g. provide electronic search strategies with population terms, clinical or health topic terms, experiential or social phenomena related terms, filters for qualitative research, and search limits).*	Page 3, lines 93-94<br>Page 4 95-98
7	**Study screening methods**	Describe the process of study screening and sifting *(e.g. title, abstract and full text review, number of independent reviewers who screened studies).*	Page 4, lines 102-108
8	**Study characteristics**	Present the characteristics of the included studies *(e.g. year of publication, country, population, number of participants, data collection, methodology, analysis, research questions).*	Page 5, lines 155-161<br>Page 6, lines 162-163
9	**Study selection results**	Identify the number of studies screened and provide reasons for study exclusion *(e,g, for comprehensive searching, provide numbers of studies screened and reasons for exclusion indicated in a figure/flowchart; for iterative searching describe reasons for study exclusion and inclusion based on modifications t the research question and/or contribution to theory development).*	Appendix S2<br><br>Page 13, lines 418-419
10	**Rationale for appraisal**	Describe the rationale and approach used to appraise the included studies or selected findings *(e.g. assessment of conduct (validity and robustness), assessment of reporting (transparency), assessment of content and utility of the findings).*	Page 4, lines 111-122
11	**Appraisal terms**	State the tools, frameworks and criteria used to appraise the studies or selected findings *(e.g. Existing tools: CASP, QARI, COREQ, Mays and Pope; reviewer developed tools; describe the domains assessed: research team, study design, data analysis and interpretations, reporting).*	Page 4, lines 111-122
12	**Appraisal process**	Indicate whether the appraisal was conducted independently by more than one reviewer and if consensus was required.	Page 4, lines 115-116
13	**Appraisal results**	Present results of the quality assessment and indicate which articles, if any, were weighted/excluded based on the assessment and give the rationale.	Table S2
14	**Data Extraction**	Indicate which sections of the primary studies were analysed and how were the data extracted from the primary studies? *(e.g. all text under the headings*	Page 4, lines 124-127

For peer review only

		"results /conclusions" were extracted electronically and entered into a computer software).	Page 5, lines 128-129
**15**	**Software**	State the computer software used, if any.	Page 4, line 102<br>Page 5, line 129
**16**	**Number of reviewers**	Identify who was involved in coding and analysis.	Page 5, line 132
**17**	**Coding**	Describe the process for coding of data *(e.g. line by line coding to search for concepts).*	Page 4, lines 134-147
**18**	**Study comparison**	Describe how were comparisons made within and across studies *(e.g. subsequent studies were coded into pre-existing concepts, and new concepts were created when deemed necessary).*	Page 5, lines 136-137
**19**	**Derivation of themes**	Explain whether the process of deriving the themes or constructs was inductive or deductive.	Page 5, lines 138-147
**20**	**Quotations**	Provide quotations from the primary studies to illustrate themes/constructs, and identify whether the quotations were participant quotations of the author's interpretation.	Page 6, lines 184-185<br>Page 7, lines 197-201<br>Page 8, lines 227-228 / 236-237 / 244-245<br>Page 9, lines 274-290<br>Page 10, lines 299-302<br>Page 11, line 331-333 / 346-351<br>Page 12, line 365 / 378-380
**21**	**Synthesis Output**	Present rich, compelling and useful results that go beyond a summary of the primary studies (e.g. *new interpretation, models of evidence, conceptual models, analytical framework, development of a new theory or construct).*	Figure 1<br>Discussion pages 13-16

For peer review only

Table S1 – Summary of Included Studies

Author (Year)	*Title*	*Sampling size and method*	*Data collection method*	*Data Analysis Method*	*Key characteristics of participants*	*Type of Loss*	*Retrospectivity of loss*	*Location*
Bansen and Stevens (1992)[52]	Women's experiences of miscarriage in early pregnancy	*n=10* Convenience sample	Open-ended interview	Content Analysis	- Mothers<br>- Aged: 23-33 (mean= 28.5 years)<br>- Ethnicity: white	Early miscarriage 6-15 weeks gestation (mean= 8.8 weeks)	2-5 months	USA
Cacciatore et al., (2008)[57]	When a baby dies: Ambiguity and stillbirth.	*n=74* Convenience sample	Focus group	Framework Analysis	- Grieving mothers, fathers, sisters, brothers, grandmothers, grandfathers and an aunt<br>- No demographic information	Stillbirth	Unknown	USA
Carlsson et al., (2016)[55]	Experiences of termination of pregnancy for fetal anomaly: A qualitative study of virtual community messages	*n= 122* Purposive sample	Cross-sectional study of messages in 2 virtual communities	Content Analysis	- Majority mothers (*n=112*)<br>- Experience of TOPFA in past year<br>- 106 (86.9%) had previous experience of TOPFA<br>- No demographic information	TOPFA after 15 weeks gestation	63% less than 1 year	Sweden
Cecil (1994)[53]	"I wouldn't have minded a wee one running about"	*n= 27* Convenience sample	Semi-structured interviews with follow up interviews	Content Analysis	- Mothers<br>- Aged 19-43<br>- Religion: Protestant (*n=13*), Catholic (*n=13*), No Religion (*n=1*)<br>- Limited demographic information	First trimester miscarriage	6 months	*UK*
Conway and Russell (2000)[54]	Couples' grief and experience of support in the aftermath of miscarriage	*n= 71* Purposive sample	Questionnaire	Content Analysis using coding frame	- Mothers (*n=39*) accompanied by partners (*n=32*)<br>-<br>- Previous children (mothers *n=22;* partners *n*=12)<br>- Socio-economic status tended towards average<br>- Limited demographic information	Miscarriage 5-16 weeks gestation (mean = 9.5 weeks)	2-4 months	*Australia*
Davis et al., (1989)[47]	Postponing Pregnancy after Perinatal Death: Perspectives on Doctor Advice	*n=24* Convenience sample	Open-ended interview	Frequency analysis	- Mothers<br>- Previous children (*n=24*)<br>- No demographic information	Stillbirth	1-10 years	*USA*
de Montigny et al., (1999)[56]	A baby has died: the impact of perinatal loss on family social networks	*n=20* Convenience sample	Open-ended questionnaire	Content analysis	- Mothers (*n=14)* and fathers (*n=6)*<br>-<br>- Experience of multiple losses: >1 perinatal death (*n=6);* 4 perinatal deaths (*n=2)*; 1 neonatal death (*n=1)*<br>- Ethnicity: white; French-speaking; middle class	Perinatal death	6 years	*Canada*

For peer review only - http://bmjopen.bmj.com/site/about/guidelines.xhtml

Author (Year)	*Title*	*Sampling size and method*	*Data collection method*	*Data Analysis Method*	*Key characteristics of participants*	*Type of Loss*	*Retrospectivity of loss*	*Location*
Grout and Romanoff (2000)[15]	The myth of the replacement child: parents' stories and practices after perinatal death	*n=10* Purposive sample	Open-ended interview	Content analysis	- Parents of 7 families (3 couples, 1 father, 3 mothers)<br>- Participants had a subsequent child between 3 and 5 years of age<br>- Experience of multiple losses (*n=3*)<br>- Previous children (*n=4*)<br>- Intact marriages<br>- Middle class; ethnicity: white	Stillbirth (including late-term miscarriage, or early infant death)	2-10 years	*USA*
Hsu et al., (2002)[50]	Transforming loss: Taiwanese women's adaption to stillbirth	n= 20 Purposive sample	Open-ended interview	Ethnographic Thematic Analysis	- Mothers<br>- Aged: 22-40 (mean = 30 years)<br>- First pregnancy (*n=6*)<br>- Previous children (*n=7*)<br>- Ethnicity: Taiwanese<br>- Limited demographic information	Stillbirth	0-2 years	*Taiwan*
Keim et al., (2017)[49]	Parent distress and the decision to have another child after an infant's death in the Neonatal Intensive Care Unit (NICU)	*n=69* Purposive sample	Semi-structured telephone interview	Content Analysis	- Mothers (*n=42*) and fathers (*n=27*) but technical difficulties meant interviews were analysed for mothers (*n=28*) and fathers (*n*=11)<br>- Aged: 18 years +<br>- Approximately 2/3 had subsequent child<br>- Majority married; majority completed secondary education<br>- Ethnicity: majority white; English speaking	Death of neonatal infant in NICU	3 months – 5 years	*USA*
Lee et al., (2013)[21]	Women's decision making and experience of subsequent pregnancy following stillbirth	*n=11* Convenience sample	Interview	Content Analysis	- Mothers<br>- No mothers had given birth to subsequent child but some were pregnant (*n=8*) (9-25 weeks (mean = 14 weeks)<br>- Aged: 26-43 (mean = 32 years)<br>- Majority first pregnancy (*n=8*)<br>- Married or cohabiting; majority in full-time work<br>- Ethnicity: white British (*n=8*), Slovakian (*n=1*), Italian (*n=1*), white Asian (*n=1*); English-speaking	Stillbirth: 24+ weeks' gestation (mean = 36 weeks)	6-12 months	*UK*

For peer review only - http://bmjopen.bmj.com/site/about/guidelines.xhtml

Author (Year)	*Title*	*Sampling size and method*	*Data collection method*	*Data Analysis Method*	*Key characteristics of participants*	*Type of Loss*	*Retrospectivity of loss*	*Location*
Meaney et al., (2017)[19]	Parents' concerns about future pregnancy after stillbirth: a qualitative study	*n=15* Purposive sample	Semi-structured interview<br><br>Couples interviewed separately	Interpretative Phenomenological Analysis	- Mothers (*n=10*) and fathers (*n=5*)<br>- Irish<br>- No demographic information	Stillbirth	4-16 months	*Ireland*
Ockhuijsen et al., (2014)[48]	Pregnancy after miscarriage: balancing between loss of control and searching for control	*n=24* Purposive sample	Semi-structured interview	Thematic Analysis	- Mothers<br>- Interviewed after 8 weeks of subsequent pregnancy. Those receiving fertility treatment were excluded<br>- Aged: 27-38 (mean=33 years)<br>- Prior experience of miscarriage 1-5 (median=2)<br>- Married or partnered; heterosexual<br>- Education ranged from secondary school to university<br>- Dutch speakers	Miscarriage	Unknown	*Netherlands*
Phipps (1986)[16]	The subsequent pregnancy after stillbirth: anticipatory parenthood in the face of uncertainty	*n=30* Purposive sample	Open-ended interview	Content Analysis	- Couples interviewed together in all but one instance<br>- Experience of stillbirth (*n=8) or* neonatal death (*n=7*)<br>- Subsequent child aged 5months – 3 years (mean = 15 months)<br>- Mother mean age: 30.2 years; Father mean age: 33.7 years<br>- Previous children (*n*=6)<br>- Homogenous, middle class sample<br>- Intact, two-parent families<br>- Ethnicity: Black (*n=1*), Hispanic (*n*=1), Caucasian (*n=13*)	Neonatal death (including stillbirth due to prematurity or congenital anomalies)	Unknown	*USA*
Tseng et al., (2014)[51]	Taiwanese women's process of recovery from stillbirth: A qualitative descriptive study	*n=21* Purposive sample	38 in-depth interview: face-to-face (*n=28*) and telephone (*n=4*)<br><br>11 participants took part in follow-up interviews	Thematic Analysis	- Mothers<br>- Aged: 23-43 (mean 31.9 years)<br>- Previous children (*n=9*)<br>- Married<br>- Employed (*n=14*)<br>- Ethnicity: Taiwanese<br>- Religion: Taiwanese folk beliefs (*n=8*), Buddhism (*n=4*), Taoism (*n=3*), Christianity (*n=2*), I-Kuan Tao (*n=2*)	Stillbirth (20-37 weeks gestation)	6 months – 6 years (mean = 2.3 years)	*Taiwan*

TOPFA – termination of pregnancy for fetal anomaly

For peer review only - http://bmjopen.bmj.com/site/about/guidelines.xhtml

Table S2: CASP Quality Assessment Results

CASP Question	Bansen and Stevens (1992)[52]	Cacciatore *et al.*, (2008)[57]	Carlsson *et al.*, (2016)[55]	Cecil (1994)[53]	Conway and Russell (2000)[54]	Davis *et al.*, (1989)[47]	de Montigny *et al.*, (1999)[56]	Grout and Romanoff (2000)[15]	Hsu *et al.*, (2002)[50]	Keim *et al.*, (2017)[49]	Lee *et al.*, (2013)[21]	Meaney *et al.*, (2017)[19]	Ockhuijsen *et al.*, (2014)[48]	Phipps (1986)[16]	Tseng *et al.*, (2014)[51]
1	Y	Y	Y	Y	Y	Y	Y	Y	Y	Y	Y	Y	Y	Y	Y
2	Y	Y	Y	Y	Y	Y	Y	Y	Y	Y	Y	Y	Y	Y	Y
3	Y	Y	Y	Y	Y	C	Y	Y	C	Y	Y	Y	Y	Y	Y
4	Y	Y	Y	Y	Y	C	Y	Y	C	Y	Y	C	Y	Y	Y
5	Y	C	Y	C	Y	C	Y	Y	C	Y	Y	Y	Y	Y	Y
6	Y	N	Y	N	N	N	N	C	Y	Y	C	N	N	C	N
7	N	N	Y	C	C	N	C	C	Y	Y	Y	Y	Y	N	Y
8	Y	N	Y	C	C	C	Y	C	C	Y	Y	Y	Y	C	Y
9	Y	N	Y	Y	Y	Y	Y	Y	Y	Y	Y	Y	Y	Y	Y
10	Y	Y	Y	N	Y	Y	Y	Y	Y	Y	Y	Y	Y	Y	Y
CASP Score	18	11	20	13	16	12	17	17	16	20	19	17	18	16	18
Quality	Good	Average	Good	Average	Good	Average	Good	Good	Good	Good	Good	Good	Good	Good	Good

Key:
Y – Yes = 2
C - Can't Tell = 1
N- No = 0

For peer review only

Table S3 – Themes and subthemes emerging from the included studies

Author (year)	Deciding about becoming pregnant		Diversity of personal reactions			Social network influences		Planning or timing subsequent pregnancy		
	Immediacy of thoughts	*Differences in reactions between parents*	*Emotional response to loss*	*Imagined family*	*Ease perinatal grief*	*Altered relationships and social networks*	*Cultural norms and expectations*	*Barriers*	*Health professional advice*	*Health professional information and reassurance*
Bansen and Stevens (1992)[52]			✓			✓	✓			✓
Cacciatore et al., (2008)[57]		✓	✓	✓		✓		✓		
Carlsson et al., (2016)[55]	✓		✓	✓	✓	✓	✓	✓		✓
Cecil (1994)[53]	✓	✓	✓				✓			
Conway and Russell (2000)[54]		✓	✓						✓	
Davis et al., (1989)[47]	✓		✓	✓	✓			✓	✓	✓
de Montigny et al., (1999)[56]			✓			✓	✓		✓	✓
Grout and Romanoff (2000)[15]			✓	✓						
Hsu et al., (2002)[50]	✓		✓	✓		✓	✓			
Keim et al., (2017)[49]	✓	✓	✓	✓	✓			✓	✓	✓
Lee et al., (2013)[21]	✓		✓	✓	✓					✓
Meaney et al., (2017)[19]	✓	✓	✓	✓		✓	✓		✓	✓
Ockhuijsen et al., (2014)[48]			✓	✓		✓	✓		✓	
Phipps (1986)[16]		✓	✓	✓		✓	✓	✓	✓	✓
Tseng et al., (2014)[51]	✓		✓	✓	✓		✓			

PRISMA 2009 Checklist

Section/topic	#	Checklist item	Reported on page #
**TITLE**			
Title	1	Identify the report as a systematic review, meta-analysis, or both.	1
**ABSTRACT**			
Structured summary	2	Provide a structured summary including, as applicable: background; objectives; data sources; study eligibility criteria, participants, and interventions; study appraisal and synthesis methods; results; limitations; conclusions and implications of key findings; systematic review registration number.	2
**INTRODUCTION**			
Rationale	3	Describe the rationale for the review in the context of what is already known.	3
Objectives	4	Provide an explicit statement of questions being addressed with reference to participants, interventions, comparisons, outcomes, and study design (PICOS).	3. SPIDER framework used in place of PICOS
**METHODS**			
Protocol and registration	5	Indicate if a review protocol exists, if and where it can be accessed (e.g., Web address), and, if available, provide registration information including registration number.	13
Eligibility criteria	6	Specify study characteristics (e.g., PICOS, length of follow-up) and report characteristics (e.g., years considered, language, publication status) used as criteria for eligibility, giving rationale.	3, 4
Information sources	7	Describe all information sources (e.g., databases with dates of coverage, contact with study authors to identify additional studies) in the search and date last searched.	4
Search	8	Present full electronic search strategy for at least one database, including any limits used, such that it could be repeated.	Appendix S1
Study selection	9	State the process for selecting studies (i.e., screening, eligibility, included in systematic review, and, if applicable, included in the meta-analysis).	4, table S1
Data collection process	10	Describe method of data extraction from reports (e.g., piloted forms, independently, in duplicate) and any processes for obtaining and confirming data from investigators.	4
Data items	11	List and define all variables for which data were sought (e.g., PICOS, funding sources) and any assumptions and simplifications made.	4
Risk of bias in individual studies	12	Describe methods used for assessing risk of bias of individual studies (including specification of whether this was done at the study or outcome level), and how this information is to be used in any data synthesis.	4, table

For peer review only

PRISMA 2009 Checklist

			S2
Summary measures	13	State the principal summary measures (e.g., risk ratio, difference in means).	n/a
Synthesis of results	14	Describe the methods of handling data and combining results of studies, if done, including measures of consistency (e.g., I^2) for each meta-analysis.	4, table S3

Section/topic	#	Checklist item	Reported on page #
Risk of bias across studies	15	Specify any assessment of risk of bias that may affect the cumulative evidence (e.g., publication bias, selective reporting within studies).	4, 11
Additional analyses	16	Describe methods of additional analyses (e.g., sensitivity or subgroup analyses, meta-regression), if done, indicating which were pre-specified.	n/a
**RESULTS**			
Study selection	17	Give numbers of studies screened, assessed for eligibility, and included in the review, with reasons for exclusions at each stage, ideally with a flow diagram.	Appendix S2
Study characteristics	18	For each study, present characteristics for which data were extracted (e.g., study size, PICOS, follow-up period) and provide the citations.	Table S1
Risk of bias within studies	19	Present data on risk of bias of each study and, if available, any outcome level assessment (see item 12).	Table S2
Results of individual studies	20	For all outcomes considered (benefits or harms), present, for each study: (a) simple summary data for each intervention group (b) effect estimates and confidence intervals, ideally with a forest plot.	n/a
Synthesis of results	21	Present results of each meta-analysis done, including confidence intervals and measures of consistency.	n/a
Risk of bias across studies	22	Present results of any assessment of risk of bias across studies (see Item 15).	n/a
Additional analysis	23	Give results of additional analyses, if done (e.g., sensitivity or subgroup analyses, meta-regression [see Item 16]).	n/a
**DISCUSSION**			
Summary of evidence	24	Summarize the main findings including the strength of evidence for each main outcome; consider their relevance to key groups (e.g., healthcare providers, users, and policy makers).	10
Limitations	25	Discuss limitations at study and outcome level (e.g., risk of bias), and at review-level (e.g., incomplete retrieval of identified research, reporting bias).	11
Conclusions	26	Provide a general interpretation of the results in the context of other evidence, and implications for future research.	12
**FUNDING**			

For peer review only

PRISMA 2009 Checklist

Funding	27	Describe sources of funding for the systematic review and other support (e.g., supply of data); role of funders for the systematic review.	13

From: Moher D, Liberati A, Tetzlaff J, Altman DG, The PRISMA Group (2009). Preferred Reporting Items for Systematic Reviews and Meta-Analyses: The PRISMA Statement. PLoS Med 6(7): e1000097. doi:10.1371/journal.pmed1000097

For more information, visit: **www.prisma-statement.org**.

For peer review only

For peer review only - http://bmjopen.bmj.com/site/about/guidelines.xhtml
